# Systematic Evaluation of Serotypes Causing Invasive Pneumococcal Disease among Children Under Five: The Pneumococcal Global Serotype Project

**DOI:** 10.1371/journal.pmed.1000348

**Published:** 2010-10-05

**Authors:** Hope L. Johnson, Maria Deloria-Knoll, Orin S. Levine, Sonia K. Stoszek, Laura Freimanis Hance, Richard Reithinger, Larry R. Muenz, Katherine L. O'Brien

**Affiliations:** 1 International Vaccine Access Center, Department of International Health, Johns Hopkins University Bloomberg School of Public Health, Baltimore, Maryland, United States of America; 2 Westat, Rockville, Maryland, United States of America; Brighton and Sussex Medical School, United Kingdom

## Abstract

**Background:**

Approximately 800,000 children die each year due to pneumococcal disease and >90% of these deaths occur in developing countries where few children have access to life-saving serotype-based vaccines. Understanding the serotype epidemiology of invasive pneumococcal disease (IPD) among children is necessary for vaccine development and introduction policies. The aim of this study was to systematically estimate the global and regional distributions of serotypes causing IPD in children <5 years of age.

**Methods and Findings:**

We systematically reviewed studies with IPD serotype data among children <5 years of age from the published literature and unpublished data provided by researchers. Studies conducted prior to pneumococcal conjugate vaccine (PCV) introduction, from 1980 to 2007, with ≥12 months of surveillance, and reporting ≥20 serotyped isolates were included. Serotype-specific proportions were pooled in a random effects meta-analysis and combined with PD incidence and mortality estimates to infer global and regional serotype-specific PD burden. Of 1,292, studies reviewed, 169 were included comprising 60,090 isolates from 70 countries. Globally and regionally, six to 11 serotypes accounted for ≥70% of IPD. Seven serotypes (1, 5, 6A, 6B, 14, 19F, 23F) were the most common globally; and based on year 2000 incidence and mortality estimates these seven serotypes accounted for >300,000 deaths in Africa and 200,000 deaths in Asia. Serotypes included in both the 10- and 13-valent PCVs accounted for 10 million cases and 600,000 deaths worldwide.

**Conclusions:**

A limited number of serotypes cause most IPD worldwide. The serotypes included in existing PCV formulations account for 49%–88% of deaths in Africa and Asia where PD morbidity and mortality are the highest, but few children have access to these life-saving vaccines.

*Please see later in the article for the Editors' Summary*

## Abstract 2

Hope Johnson and colleagues calculate the global and regional burden of serotype-specific pneumococcal disease in children under the age of five.

## Editors' Summary

**Background:**

Despite all the international attention on Millennium Development Goal (MDG) 4—to reduce deaths in children under 5 years by two thirds by 2015—pneumonia, sepsis, and meningitis together comprise >25% of the 10 million deaths occurring annually in children <5 years of age. *Streptococcus pneumoniae* is a leading bacterial cause of these diseases and the World Health Organization estimates that approximately 800,000 children die each year of invasive pneumococcal disease. Three pneumococcal conjugate vaccines are currently available and protect against the serotypes most commonly causing invasive pneumococcal disease in young children in North America. However, few countries with the highest burden of invasive pneumococcal disease have introduced the vaccines into their national immunization programs. Not only is it important to introduce a vaccine, but also to use a vaccine covering the appropriate serotypes prevalent in a susceptible region.

**Why Was This Study Done?:**

Over the past few years, data on serotyping in many high burden countries has become available. The authors conducted this study (a systematic review and meta-analysis) to quantify the serotypes causing invasive pneumococcal disease in children <5 years of age in order to estimate the global and regional serotype distribution and serotype-specific disease burden. This information can then be used to estimate the potential public health impact of pneumococcal conjugate vaccine formulations and help to inform decision making for both pneumococcal vaccine development and the introduction of a vaccine into a specific region.

**What Did the Researchers Do and Find?:**

Using published studies and unpublished data provided by researchers, the researchers systematically reviewed studies that included data on invasive pneumococcal disease serotype among children <5 years of age. The researchers then used statistical tools to pool the serotype-specific proportions and combined this information with pneumococcal disease incidence and mortality estimates to calculate the global and regional burden of serotype-specific pneumococcal disease.

The researchers reviewed 1,292 studies and included 169 suitable studies in their analysis, which included information on 60,090 isolates from 70 countries. The researchers produced regional estimates of the serotypes that caused invasive pneumococcal disease among under five-year-olds in different regions: six serotypes were identified as causing most invasive pneumococcal disease in North America; nine serotypes were identified in Africa; and 11 serotypes were identified in Asia. The researchers also found that seven serotypes (1, 5, 6A, 6B, 14, 19F, and 23F) were the most common globally and that these seven serotypes accounted for 58%–66% of invasive pneumococcal disease in every region. On the basis of incidence and mortality estimates of invasive pneumococcal disease for the year 2000 (before pneumococcal conjugate vaccines were introduced), the researchers found that these serotypes represented >300,000 deaths in Africa and 200,000 deaths in Asia.

**What Do These Findings Mean?:**

This study shows that a limited number of serotypes cause most invasive pneumococcal disease worldwide. This finding contradicts the conventional supposition that the most common serotypes causing invasive pneumococcal disease vary greatly across geographic regions. Crucially, the findings of this study also show that the serotypes currently included in existing pneumococcal conjugate formulations account for 49%–74% of deaths in Africa and Asia where the morbidity and mortality of pneumococcal disease are the highest and where most children do not have access to current pneumococcal conjugate vaccines. Although the authors do not provide country-level estimates of serotype distribution, country-specific vaccine impact estimates can be made using country-level pneumococcal disease burden numbers combined with the regional serotype estimates provided in this study. This means that national policy makers can assess the potential impact of serotypes included in different conjugate vaccines, which should contribute to their decision-making process. In addition, manufacturers can now work from a consensus set of serotype coverage estimates to plan and design future serotype-based vaccine formulations to target the pneumococcal disease burden.

**Additional Information:**

Please access these Web sites via the online version of this summary at http://dx.doi.org/10.1371/journal.pmed.1000348

- The World Health Organization provides information about pneumococcus
- The PneumoACTION provides information about pneumonia and pneumococcal disease
- The Global Alliance for Vaccination and Immunisation has information on all aspects of vaccination and immunization
- The US Centers for Disease Control provides information about pneumococcal conjugate vaccination
- The Word Pneumonia Day coalition provides information about pneumonia

## Introduction

Pneumonia, sepsis, and meningitis together comprise >25% of the 10 million deaths that occur in children <5 y of age worldwide [1]. *Streptococcus pneumoniae* (SP, pneumococcus) is the leading bacterial cause of these diseases. The World Health Organization (WHO) estimates that approximately 800,000 children die each year of pneumococcal disease (PD), and 90% of these deaths occur in developing countries [2].

Three pneumococcal conjugate vaccines (PCVs) are currently available and are protective in young children. A 7-valent vaccine (PCV7, Prevnar, Pfizer) was licensed in 2000 and contains SP serotypes 4, 6B, 9V, 14, 18C, 19F, 23F—the serotypes most commonly causing invasive PD (IPD) among young children in North America (NA). PCV10 (Synflorix, GlaxoSmithKline) was licensed for use in Canada, Australia, and Europe in late 2008 to early 2009 and contains PCV7 serotypes plus serotypes 1, 5, and 7F. PCV13 (Prevnar13, Pfizer) added serotypes 3, 6A, and 19A to the PCV10 serotypes and was licensed in Chile and by the European Medicines Agency in 2009. PCV7 is currently being replaced by PCV13 vaccine as manufacturing and supply are scaled up. Both PCV10 and PCV13 are eligible for the Pneumococcal Advance Market Commitment (AMC) and both manufacturers have agreed to supply Global Alliance for Vaccines and Immunisation (GAVI) Alliance–eligible countries as part of the AMC.

Despite the availability of PCV7 since 2000, few countries in geographic regions with the highest burden of pneumococcal disease have introduced the vaccine into national immunization programs. In October 2006, WHO convened an expert consultation meeting to evaluate the minimum or optimal serotype composition of PCVs for use in resource-poor countries. Estimates of serogroup distributions were published in year 2000 by Hausdorff et al., but since then, additional data have become available on serotypes for countries in regions where disease burden is the highest [3]. After reviewing IPD epidemiology and existing literature on the global distribution of serotypes [3], the expert group concluded that updated estimates of serotype distribution were needed [4].

In February 2007, the GAVI Alliance announced a US$1.5 billion Pneumococcal AMC, to fund a novel vaccine-financing mechanism to accelerate the development and introduction of pneumococcal vaccines for the world's poorest countries (GAVI Alliance–eligible: countries with a gross national income per capita <US$1,000 in year 2003). Essential to the Pneumococcal AMC was the establishment of minimum vaccine product specifications, also known as the target product profile (TPP), for a vaccine to be eligible for AMC funds. Here we report the systematic review and meta-analysis of serotypes causing IPD in children <5 y as part of the Pneumococcal Global Serotype Project (GSP), which estimated global and regional serotype distribution, serotype-specific disease burden, and the potential public health impact of PCV formulations to inform decision making regarding pneumococcal vaccine development and introduction.

## Methods

We performed a systematic review of the published literature and meta-analysis using a protocol developed by the authors and summarized below.

### Human Research

This research was not considered human participants research and therefore did not require approval by an Institutional Review Board.

### Sources of Data

We identified articles published as of July 15, 2007 with serotype data pre-PCV introduction from IPD cases (defined as isolation of SP from a normally sterile site) among children <5 y by systematically searching 12 literature databases (PubMed, Medline, Embase, Global Health Database, Biosis, PASCAL, Current Contents, African Index Medicus, Index Medicus for the WHO Eastern Mediterranean Region [IMEMR], Latin American and Caribbean Health Sciences Information (LILACS), Health Literature, Library and Information Services (HELLIS), which also contains Index Medicus for the South East Asia Region [IMSEAR]), modifying established keywords and search terms described elsewhere (Table S1) [5]. The reference lists of retrieved papers were searched for further studies. We also contacted researchers known to carry out IPD surveillance and requested supplemental and unpublished serotype data, some of which became available in the public domain after July 15, 2007. Non-English language articles were included and extracted by abstractors proficient in the relevant languages.

### Inclusion Criteria

We included studies reporting at least 20 serotyped pneumococcal isolates obtained from normally sterile sites (e.g., blood, cerebrospinal fluid, pleural fluid) conducted since year 1980 with a duration of at least 12 mo of surveillance due to the seasonal nature of PD and, for the US, Canada, Australia, and Europe, pre-PCV7 national introduction. Where possible, data were limited to children <5 y of age, excluding neonates. However, data were frequently presented in aggregate with older children and we attempted to contact every author to obtain serotype data limited to children <5 y of age. In consultation with our External Expert Committee (EEC), who provided technical review and advice on our study methods and interpretation of analyses, we decided to include studies with serotype data from children age ≥60 mo if data were presented in aggregate with serotype data from children age 0–59 mo in order to improve geographic representativeness of the serotype data included in the analysis, particularly in high disease-burden regions. We assumed most cases in these studies occurred among children ≤5 y of age and included studies in NA and Europe through 83 mo of age consistent with epidemiology of pneumococcal disease. For all other countries and regions where data were less plentiful, we included studies with aggregated serotype data through 215 mo of age. Additional serotype data from children were available from countries but not included in the analysis if the serotype data were presented in aggregate with children older than these respective age cutoffs (i.e., 83 mo of age in NA and Europe and 215 mo of age in all other geographic regions). Studies testing or reporting only serotypes included in existing vaccine formulations (e.g., only reported PCV7 vaccine serotype coverage) were excluded from the analysis because of the potential bias of underreporting nonvaccine serotypes.

### Data Collection and Management

Two reviewers abstracted each study using a pretested paper form. Data abstracted included study design and setting, and the proportion of IPD caused by each serotype stratified by age, syndrome (i.e., meningitis, pneumonia, nonpneumonia/nonmeningitis, e.g., bacteremia without a focus, and IPD not otherwise specified), specimen type, and HIV status. Definition of syndrome was based on author report and no attempt was made to standardize definitions across studies. Discrepant data were reabstracted by a third reviewer whose abstraction was considered final. Data were double-entered into a customized Microsoft Access database (Microsoft), which included several data checks to ensure completeness and accuracy of the data abstracted and entered.

### Data Analysis

The proportion of IPD due to each serotype was estimated independent of the other serotypes using marginal random-effects meta-analysis within geographic regions defined according to United Nations definitions to estimate regional serotype distributions [6],[7]. Serogrouped isolates that were not further subtyped were apportioned into serotypes by applying the regional serotype estimates for that serogroup from the current analysis. A small nonzero value (0.5 isolates to the numerator and denominator) was added to all observations to accommodate serotypes reported as 0% whose study weights (the inverse of the variance) would otherwise be undefined. To minimize the resulting bias introduced, estimation was limited to only 21 serotypes and an aggregate “all other serotypes” category. The selection of serotypes to be modeled was based on the top 20 most common serotypes globally after adjusting for regional PD incidence in a preliminary analysis restricted to studies with all isolates serotyped (Table S2). The serotypes identified as the top 20 globally included the PCV13 serotypes and the 13 most common serotypes in each region with the exception of serotype 9A. Serotype 9A was among the top 13 ranked serotypes in NA but not among the 20 most common global serotypes (ranked 25th), thus it was added as the 21st serotype to be estimated. To normalize serotype estimates so they sum to 100%, each independently estimated serotype proportion was divided by the sum of the proportions. The uncertainty range for each serotype proportion was estimated by the 95% confidence interval (CI) from the meta-analysis and lower bounds were set to zero if negative. The same statistical methods were applied to provide estimates using other categories for stratified analysis including serotype distribution by region excluding the single country with the greatest number of isolates in the regional analysis, by GAVI Alliance eligibility, and distribution of serotypes by age group.

Concordance of the serotype proportions observed among individual studies used within a regional analysis was examined by calculating the intraclass correlation (ICC) for each serotype [8]. When the ICC is high (i.e., informally defined as between 0.7 and 1.0), the variability in serotype proportion between studies is considered small compared to the natural statistical variation within a study (i.e., within-study variance). By contrast, when the ICC is low (i.e., <0.5), different studies are as, or more, variable in their reporting of a given serotype proportion than the natural statistical variation within a study.

To estimate the global serotype distribution, regional serotype distributions were weighted by WHO global burden of disease estimates of regional incidence of PD cases (GBD Project) [2]. Serotype-specific PD incidence and mortality were estimated by multiplying the regional serotype distributions by the GBD Project incidence and mortality estimates [2]. Identical serotype distribution for both bacteremic and nonbacteremic PD was assumed for both cases and deaths. To estimate the uncertainty, we multiplied the upper and lower bounds of the 95% CI for each serotype proportion by the upper and lower uncertainty bounds from the GBD Project estimates [2].

PCV7, PCV10, and PCV13 serotype coverage was defined as the sum of the vaccine-type serotype proportion meta-analysis estimates with 95% CIs estimated using bootstrapping methodology [9]. We assumed serotype 6A/6B cross-protection for PCV7 and PCV10 on the basis of evidence from PCV efficacy trials and postvaccine introduction impact data from the US Centers for Disease Control and Prevention Active Bacterial Core surveillance [10]–[12]. To estimate the burden of disease associated with specific PCV formulations, the vaccine serotype coverage for IPD was multiplied by the GBD Project incidence and mortality estimates for PD [2].

Statistical analyses were conducted in SAS (Version 9.1.3, SAS Institute) and validated by a second statistical programmer. An EEC and *ad hoc* consultations with other relevant experts provided technical review and advice on the study methods and interpretation of analyses.

## Results

### Data Reviewed

We identified 1,232 published articles and reports and researchers provided unpublished data from an additional 60 studies (Figure S1; Text S1). We retrieved the full text of 618 articles after title and abstract screening and removal of duplicate data (*n* = 44). Full-text screening excluded studies without SP isolated from a sterile site (*n* = 139), or serotype data (*n* = 161), or if reported serotype data were not abstractable for study purposes (e.g., presented in aggregate with age group/specimens not meeting inclusion criteria) (*n* = 149).

169 studies were included in the final analysis, representing 70 countries and 60,090 serotyped isolates (Table 1). Every geographic region contributed a substantial number of studies (range: 16–42) and isolates (range: 3,649–18,788), with the fewest number of isolates from Oceania (3,649) and Asia (4,752). The number of isolates from studies included in the analysis range from 20 to 8,221, and most studies in Africa, Asia, and Europe reported serotype data for less than 100 IPD cases. Studies typically report a smaller number of serotyped isolates if the surveillance (a) has less sensitive detection methods, (b) is of shorter duration, or (c) does not serotype isolates from all identified cases. Half of the serotype data in the analysis (from 42/169 studies) was either unpublished or supplemental to published data obtained from contacted authors (Table S3) and review of these data enabled abstraction of serotype data for all serotypes tested for, in contrast to published data in which serotype data for less common serotypes were frequently not reported.

**Table 1:** Summary of serotype data available for analysis.

Serotype Data	Category	Africa	Asia	Europe	LAC	NA	Oceania	Total (Global)
*n* Countries (percent of countries in region)	—	13/53 (24.5)	18/47 (38.3)	16/42 (38.1)	17/33 (51.5)	2/2 (100.0)	4/16 (15.0)	70/193 (36.3)
*n* GAVI-eligible countries (percent of countries in region)	—	10/39 (25.6)	6/22 (27.3)	0/2 (0.0)	3/6 (50.0)	0/0 (0.0)	3/3 (100.0)	20/72 (27.8)
*n* Studies	—	22	33	39	42	17	16	169
*n* Studies by *n* serotyped isolates in study (percent of studies in region):	20–49	7 (31.8)	7 (21.2)	13 (33.3)	4 (9.5)	2 (11.8)	4 (25.0)	37 (21.9)
	50–99	4 (18.2)	11 (33.3)	9 (23.1)	8 (19.1)	2 (11.8)	2 (12.5)	36 (21.3)
	100–199	6 (27.3)	9 (27.3)	5 (12.8)	10 (23.8)	2 (11.8)	5 (31.3)	37 (21.9)
	200–499	2 (9.1)	5 (15.2)	8 (20.5)	9 (21.4)	6 (35.3)	1 (6.3)	31 (18.3)
	≥500	3 (13.6)	1 (3.0)	4 (10.3)	11 (26.2)	5 (29.4)	4 (25.0)	28 (16.6)
*n* Studies with serotype data stratified for children age <2 y and ≥2 y (percent of studies in region)		11/22 (50.0)	8/33 (24.2)	12/39 (23.1)	4/42 (9.5)	11/17 (64.7)	6/16 (37.5)	49/169 (29.0)
*n* Studies by syndrome (percent of studies in region):	Meningitis Only	3/22 (13.6)	3/33 (9.1)	3/39 (7.7)	5/42 (11.9)	0/17 (0.0)	0/16 (0.0)	14/169 (8.3)
	Nonmeningitis only	1/22 (4.6)	3/33 (9.1)	4/39 (10.3)	1/42 (2.4)	1/17 (5.9)	1/16 (6.2)	11/169 (6.5)
	Mixed^a^	18/22 (81.8)	27/33 (90.8)	32/39 (82.0)	36/42 (85.7)	16/17 (94.1)	15/16 (93.8)	144/169 (85.2)
*n* Isolates	—	11,181	4,752	10,279	18,788	11,441	3,649	60,090
*n* Isolates per 100,000 children <5 y in year 2000	—	9	1	28	34	53	136	9

a Studies with cases from any of the following syndromes: meningitis, pneumonia, nonpneumonia/nonmeningitis (e.g., bacteremia without a focus), invasive disease not specified/unknown.

### Regional Serotype Distribution

**Figure 1:**
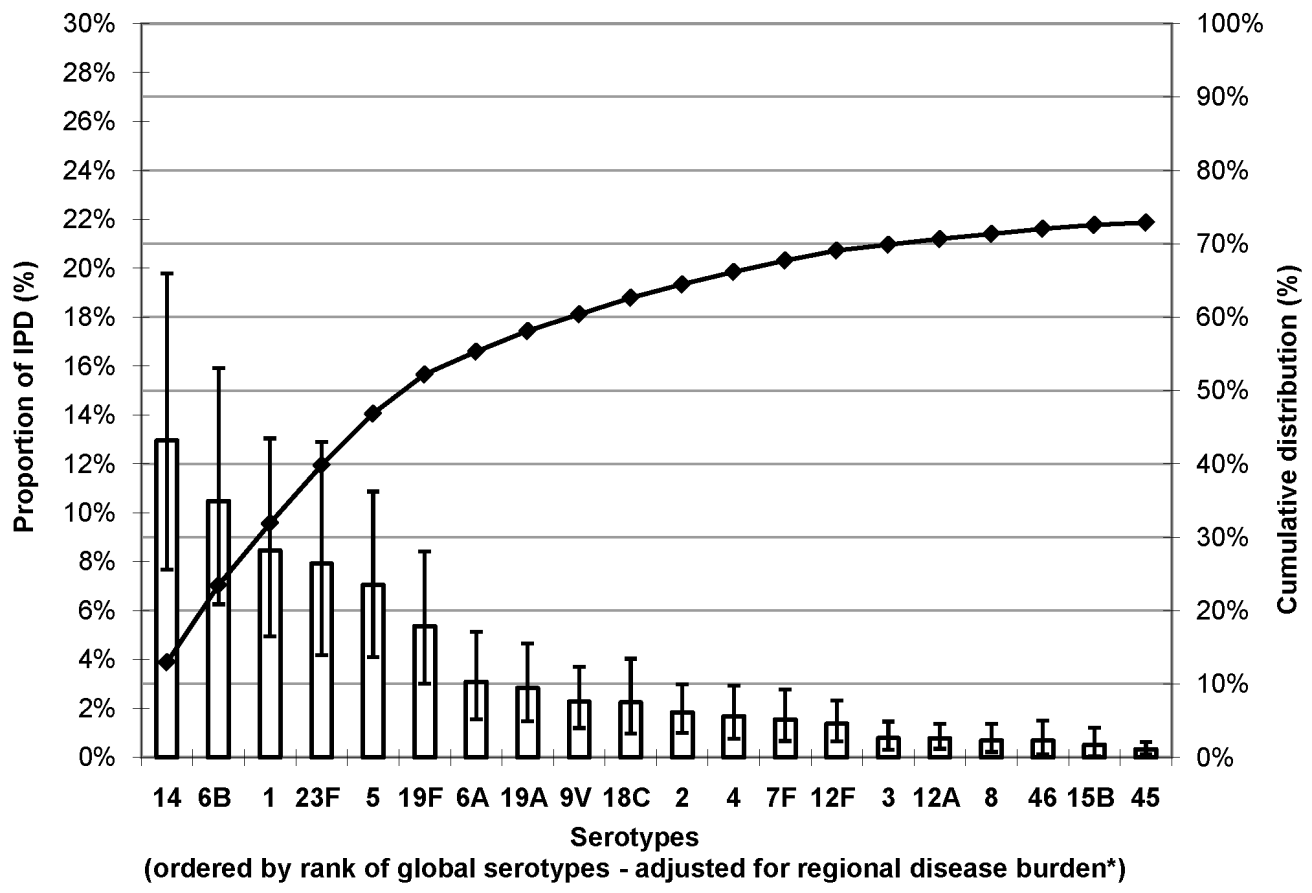
Proportion of IPD in young children due to the most common serotypes globally. Error bars indicate the 95% CI for the proportion of invasive PD due to each of the 21 serotypes. Cumulative line indicates the cumulative proportion of invasive PD due to the 21 serotypes. *Adjusted for regional incidence of cases.

Regional estimates of the proportion of IPD among young children showed that a limited number of serotypes seem to cause most IPD worldwide (Figure S2). The number of serotypes accounting for ≥70% of IPD ranged from 6 in NA (95% CI 5–7) to 9 in Africa (95% CI 8–11) and 11 in Asia (95% CI 9–13) (Figure S3). Seven serotypes (1, 5, 6A, 6B, 14, 19F, and 23F) were the most common globally (Figure 1), the seven most common in both Africa and Asia, and accounted for 58%–66% of IPD in every region (Figure S2). Each region had a single country with a significantly greater number of isolates than any other country in that region (i.e., Asia, Israel; Africa, South Africa; Latin America and Caribbean (LAC), Brazil; NA, US; Europe, United Kingdom: and Oceania, Australia) (Figure S4). Excluding isolates from the single country with the greatest number of isolates in the regional analysis does not substantively change distributions of the African and Asian serotypes (Figure S5).

Serotype 14 was the most common serotype accounting for 12%–29% of IPD in each region. Serotype 6B ranked second in every region, except Africa (ranked fifth); when combined with serotype 6A, this serogroup accounted for 14%–18% of IPD across regions. Serotype 6C, which was identified in 2007 [13],[14], was not reported in the studies included in this analysis. Serotype 1, a known cause of meningitis outbreaks in the African meningitis belt [15], ranked among the top four serotypes in those regions with the highest IPD burden (Africa, Asia, and LAC). Serotype 5 ranked third in Africa and LAC, and fifth in Asia. Serotypes 1, 5, and 14 together accounted for 28%–43% of IPD across regions. Serotypes 23F and 19F were responsible for 9%–18% of IPD overall. Serotype 18C was common (ranked fourth or fifth) in regions with a large proportion of high income countries (i.e., Europe, NA, and Oceania). Serotype 19A, which is frequently associated with antibiotic resistance [16], was relatively more important in Europe (6%) than other regions (range: 3%–4%).

The heterogeneity in reported serotype prevalence varied by serotype and region (Table S4). The top-ranked serotypes in regions with the highest burden of pneumococcal disease (Africa, Asia, and LAC) have low ICCs indicating high variability across studies in the reported proportion of IPD due to these serotypes. In geographic regions with low ICCs for the reported serotypes, the addition or removal of studies has a large impact on the overall regional meta-estimate for those serotypes. In Africa, ten serotypes had an ICC<0.2, thus the meta-estimates for these ten serotypes are particularly sensitive to the addition or removal of serotype data in this region. Asia and LAC also had a large number of serotypes with ICC<0.2 (six and five serotypes, respectively). By contrast, in Oceania, only serotype 14 had an ICC<0.2, indicating relative homogeneity in the reported serotype proportion data for this region. Higher ICC values for less common serotypes may reflect the bias in underreporting of less common serotypes in published studies, or may be due, in part, to the methods used in this analysis, including inclusion of studies with a small number of serotyped isolates that are unable to estimate the proportion of IPD caused by less prevalent serotypes; and, further, the addition of a small nonzero value to serotype proportions to accommodate serotypes reported as 0% whose study weights would otherwise be undefined, which has a greater impact on the estimated proportion of IPD due to less common serotypes.

### Serotype Distribution by GAVI Alliance Eligibility

**Figure 2:**
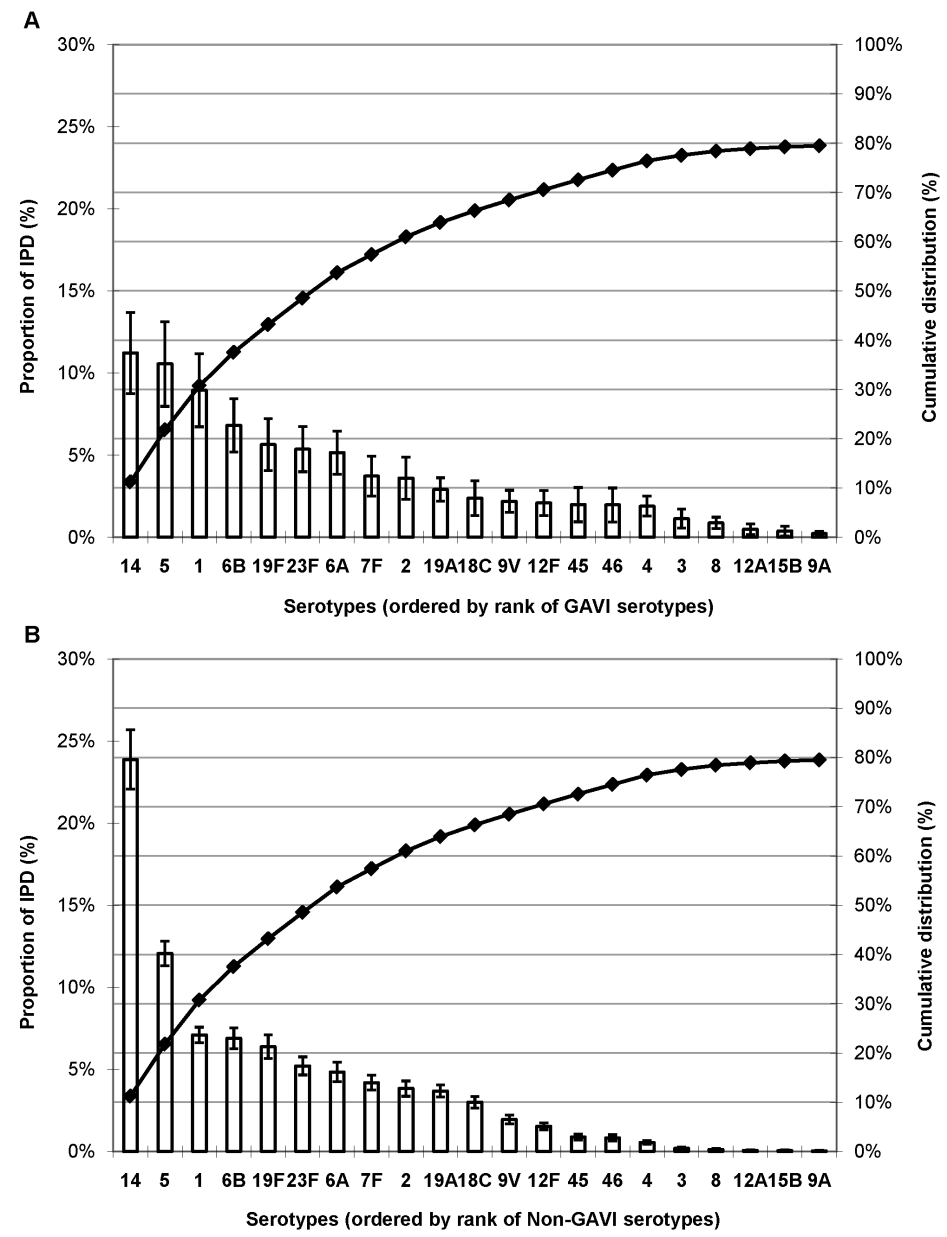
Proportion of IPD in young children due to the 21 most common or important serotypes by GAVI Alliance eligibility. (A) GAVI Alliance–eligible countries and (B) non-GAVI Alliance eligible countries. Error bars indicate the 95% CIs. Line indicates the cumulative proportion across serotypes.

Serotype data (4,678 isolates) available from 20 of the world's 72 poorest countries (GAVI Alliance–eligible countries) showed that serotypes 14, 5, and 1 were the most common serotypes accounting for >30% of IPD (Figure 2). In non-GAVI Alliance–eligible countries, serotype 14 caused most (24%) IPD, and serotypes 1 and 5 ranked fifth and eighth, respectively. Serotypes 6B, 19F, and 23F were the next most commonly observed serotypes for both GAVI Alliance- and non-GAVI Alliance–eligible countries.

### Serotype Distribution by Age Group

**Figure 3:**
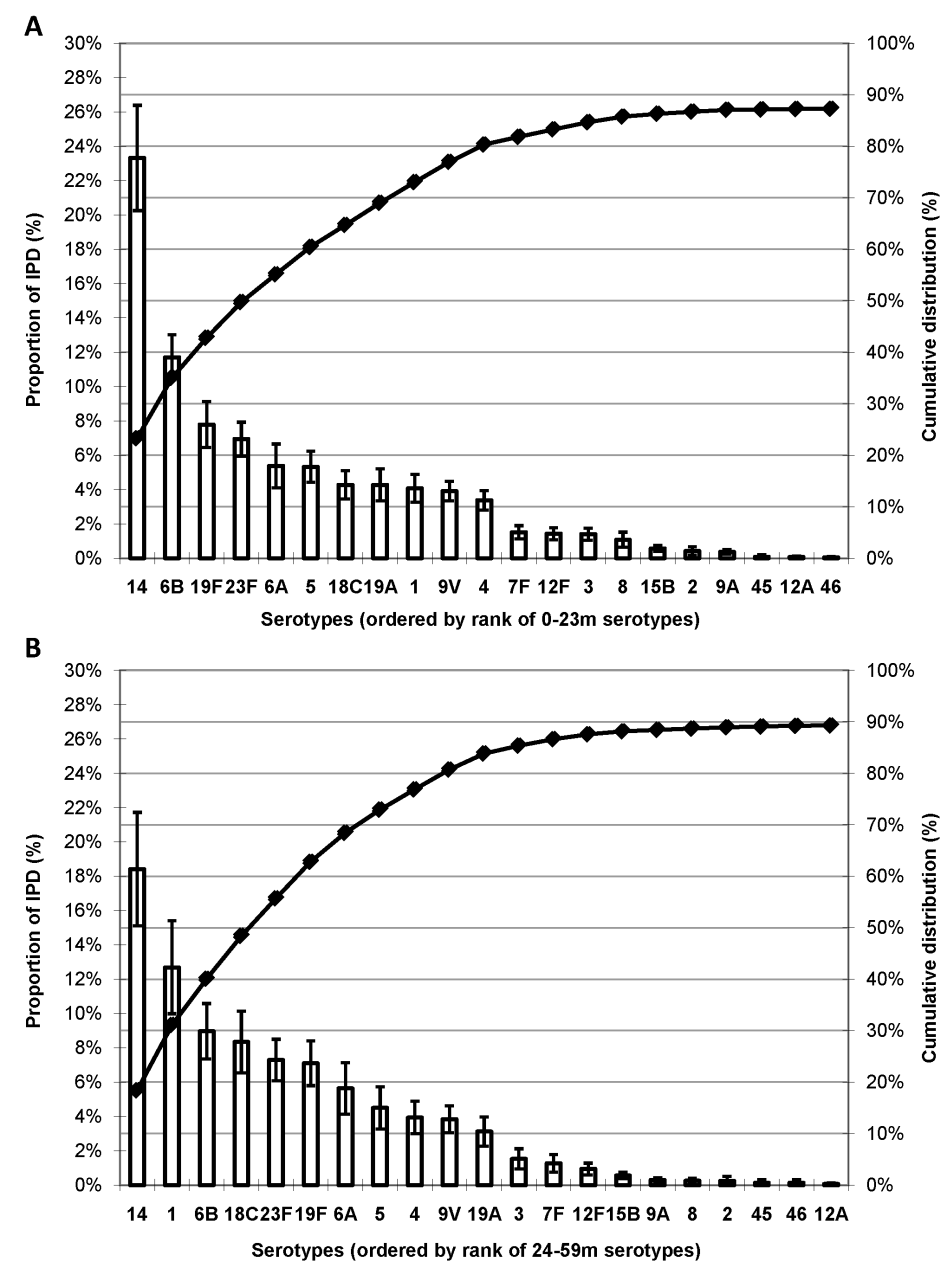
Proportion of IPD in young children globally due to the most common serotypes by age. (A) children age 0–23 mo (*n* = 18,829 isolates) and (B) children age 24–59 mo (*n* = 6,664 isolates). Error bars indicate the 95% CIs. Line indicates the cumulative proportion of IPD across serotypes. *Limited to studies (*n* = 49) with serotype data available for both age groups.

In 49 studies with serotype data available for both <2- and ≥2-y-old age groups, approximately 26% of the 25,493 isolates were from children ≥2 y of age. While serotype 14 dominated the global serotype distribution for both age groups, serotype 1 was less common among children <2 y of age (ranked ninth) than among 2–4 year olds (ranked second; Figure 3); similar findings were observed in each region (unpublished data).

### Serotype-Specific Disease Burden

Based on the year 2000 WHO GBD Project incidence and mortality estimates, the seven global serotypes (1, 5, 6A, 6B, 14, 19F, and 23F) account for approximately 9 million cases and 500,000 deaths due to PD in children <5 y of age. In areas of high PD incidence (i.e., Africa, Asia, LAC, Oceania), the incidence accounted for by the three most common serotypes exceeded the total PD incidence and mortality rate in NA and Europe (Tables S6, S7, S8, S9).

### Vaccine Serotype Coverage and Potential Health Impact

**Figure 4:**
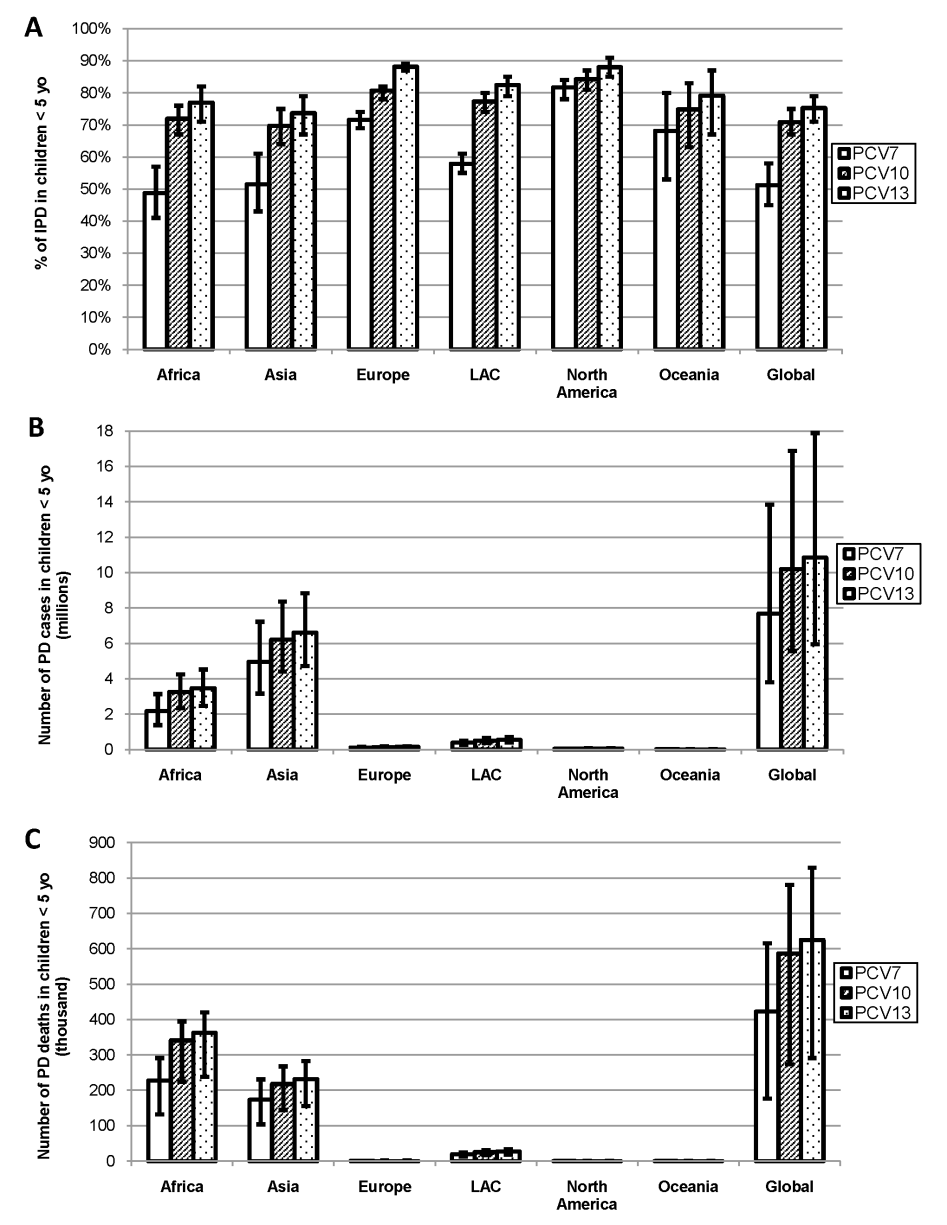
(A) Proportion of IPD, number of (B) PD cases, and (C) deaths in children <5 y of age due to serotypes in existing PCV formulations. Assumes serotype 6A/6B cross-protection, globally and by region. Error bars indicate the 95% CIs (A) or uncertainty estimates (B, C). PCV7 serotypes include: 4, 6B, 9V, 14, 18C, 19F, 23F. PCV10 adds serotypes: 1, 5, and 7F. PCV13 adds serotypes: 3, 6A, and 19A.

Serotypes included in PCV7 accounted for ≥49% of IPD in each region, but this percentage varied substantially by region (range: 49%–82%), with highest serotype coverage in NA and Europe (Figure 4A). Coverage of PCV10 was similar to PCV13, with serotypes accounting for ≥70% of IPD in every region and less regional variability (range: 70%–84% and 74%–88%, respectively) than with PCV7 (Figures 4, and S6, S7, S8). Globally, PCV7 serotypes accounted for approximately 7.4 million cases and 400,000 deaths due to PD in children <5 y of age (Figure 4B, 4C). The additional serotypes in PCV10 and PCV13 accounted for another 2.8 and 3.4 million PD cases and 170,000 and 200,000 PD deaths, respectively.

## Discussion

Our findings contradict the conventional supposition that the most common serotypes causing IPD vary greatly across geographic regions. Serotype 14 was the most common cause of IPD in every region, and seven serotypes (1, 5, 6A, 6B, 14, 19F, 23F) accounted for more than half of IPD in every region. Serotype 1 was an important cause of IPD in Africa, Asia, and in Europe, contravening the widely held notion that this serotype is only common in developing country settings [4]. Although we identified seven serotypes common across regions, the proportion of IPD caused by these serotypes varied across and within regions. The serotypes included in existing PCVs are responsible for a large portion of IPD in every region; PCV10 and PCV13 provide considerably greater coverage than PCV7 in regions with the largest PD burden. Serotypes not included in existing vaccine formulations are less common, therefore serotype-based vaccine formulations developed to extend valency beyond the existing PCV13 may only confer a marginal additional serotype coverage and may be more complex and costly to manufacture.

Our region-specific estimates of vaccine serotype coverage are consistent with those provided elsewhere [3],[10],[16]. We estimated 82% (95% CI 78%–84%) PCV7 serotype coverage (assuming serotype 6A/6B cross-protection) for NA, which is consistent with the observed 78% (95% CI 74%–82%) reduction in IPD vaccine and vaccine-related serotypes post-PCV7 introduction in the US before significant herd effects were seen [10]; and, similar to previous estimates, PCV7 serotype coverage in NA is approximately 10% greater than for Europe [3].

There were notable differences in our results compared with those from the year 2000 analysis by Hausdorff et al. [3], which may be attributable to differences in the included studies, availability of serotype versus serogroup data, and the statistical modeling methods used (random versus fixed-effects meta-analysis in Hausdorff et al.). The serotype analysis presented here includes more than double the number of studies, isolates, and data from developing countries than the analysis by Hausdorff et al. [3]. The number of isolates that were serogrouped and not further subtyped in the present analysis were few (6.7% of all isolates) and were typically more common serogroups (6, 18, 19, 23) for which robust subtype data were available at the regional level. Because of the lack of available serotype data, Hausdorff et al. provided estimates of serogroup distribution only, and therefore estimates of vaccine serotype coverage were less specific. Hausdorff et al. found lower PCV7 serotype coverage in Asia (∼30%) compared to our study (∼50%), and that the important serotypes in Africa differed from those in Asia [3], whereas we found that the seven most common serotypes in Africa were identical to those in Asia, albeit the proportion of IPD caused by those serotypes varied.

A limitation of the review by Hausdorff et al. and the present analysis is the inclusion of isolates from children age ≥60 mo in the analysis that estimates serotype distribution for young children (see Table S10). Serotype 1 is more important in older children (as seen in Figure 3; additional unpublished data), thus it is possible that the estimates of proportion of IPD caused by serotype 1 are slightly overestimated, but the effect is likely minimal due to the lower incidence of IPD in this age group. Additional limitations include those common in analyses of published data: (a) data are often presented in aggregate without further specification (i.e., in “other”); (b) data stratified by categories are inconsistent with variables of interest thus limiting the availability of data for stratified analyses (e.g., serotype distribution by HIV status and syndrome); and (c) difficulty in interpreting missing serotype data as either not tested for, or tested for and not reported, or tested for and grouped in “other.” Analysis of syndrome-specific serotype distribution is confounded by age and therefore not reported here but multivariable analyses using large datasets with case-level data are currently underway and expected to provide further insights into serotype distribution differences by age, syndrome, and HIV status.

Several features of the serotype data limited the feasibility of more sophisticated statistical methods. We abstracted and used the serotype proportions reported in each study in the analysis and could also have analyzed logit transformations of the proportions, but this would likely only impact estimates for less prevalent serotypes for which few studies included a sufficient number of isolates to be able to estimate the proportion of IPD caused by less common serotypes. Compositional multivariate statistical methods such as multinomial logistic regression were preferred but not feasible due to the large number of serotypes (i.e., categories) estimated, because different serotypes were reported across studies, and, because it was difficult to interpret missing serotype data as mentioned previously. Additionally, we used simple (single) rather than multiple imputation to apportion serogroup data to subtypes, which could have underestimated the variability in serotype proportions, but since only 6.7% of all isolates included in the analysis were not subtyped the effect should be minimal.

Owing to the lack of serotype-specific incidence and mortality data available in the published literature, our estimates relied on serotype prevalence among cases of IPD estimated from the literature applied to existing pneumococcal morbidity and mortality estimates. Estimates of serotype-specific incidence assumed the same serotype distribution for both bacteremic and nonbacteremic disease (i.e., IPD and PD); and serotype-specific mortality assumed uniform risk of mortality for each serotype, which may not be entirely correct, therefore serotype-specific estimates of morbidity and mortality may be slightly over- or underestimated for individual serotypes.

A strength of the present analysis is the inclusion of a large number of studies and isolates covering more than 20 y allowing for a “smoothing” of secular trends and small studies to estimate the average serotype distribution causing PD globally and regionally over time. Secular trends and outbreaks of serotype distribution are known to occur [17]–[19]. Studies of short duration risk over- or underestimating serotype coverage due to inability to take into account the periodicity of serotypes.

Serotype data from a large number of countries also strengthen the regional estimates. Although limited or no data are included from countries with the largest populations of children <5 y of age (e.g., mainland China and India each contributed <250 isolates, and no isolates were included from Indonesia and Nigeria), serotype data from neighboring countries (e.g., Pakistan, Bangladesh, Papua New Guinea, Taiwan, Hong Kong, Ghana, Burkina Faso) are included in this analysis and ensure some representation of data from these subregions. While additional data might allow for generation of subregional estimates and an assessment of the effects of age, syndrome, HIV status, or other factors on the distribution of serotypes, it is unlikely that additional pre-PCV introduction serotype data would significantly change the regional inferences of this analysis (Figure S5). However, continued surveillance is needed to monitor trends in serotype distribution post-PCV introduction to evaluate the impact and value of specific vaccines and immunization strategies.

The large amount of serotype data available for this analysis reflects an increase in awareness and support for surveillance of bacterial pneumonia and meningitis [20]–[24]. In addition to estimates of vaccine serotype coverage, this is the first report, to our knowledge, of global estimates of serotype-specific PD incidence and mortality, allowing us to project the potential public health impact of existing PCV formulations. With advancements in vaccine development and financing mechanisms for vaccine procurement, estimates of disease burden and serotype distribution are essential to inform decision making for vaccine introduction and to monitor vaccine impact.

PD is the leading vaccine-preventable disease among children <5 y of age globally [25]. Until recently, countries with high PD incidence and mortality have not had access to life-saving pneumococcal vaccines. In April 2009, Rwanda was the first GAVI Alliance–eligible country to introduce PCV into routine childhood immunizations. Other countries are contemplating or preparing policy analyses of PCV products. Although we do not provide country-level estimates of serotype distribution, country-specific vaccine impact estimates can be made using country-level PD burden numbers combined with the regional serotype estimates provided here [2]. Use of these regional serotype prevalence estimates in vaccine impact calculations and cost-effectiveness analyses are underway and will provide country-level policy makers with estimates of the potential impact of various PCV formulations, which should contribute substantially to the decision-making process. Likewise manufacturers can work from a consensus set of serotype coverage estimates to plan and design future serotype-based vaccine formulations targeting the greatest pneumococcal disease burden. Recent progress towards increasing access to PCVs in high-burden countries will contribute to achieving the year 2015 Millennium Development Goal 4 target to reduce child mortality by two-thirds [26].

## Supporting Information

Figure S1 **Flow chart of study identification.** Details of excluded studies can be found in the text. *Other sources of data include unpublished data received from contacted researchers.
(0.07 MB DOC)

Figure S2 **Proportion of IPD in young children due to the 21 most common or important serotypes by region.** (A) Africa, (B) Asia, (C) Europe, (D) LAC, (E) NA, (F) Oceania. Error bars indicate the 95% CIs. Line indicates the cumulative proportion across serotypes [27].
(0.51 MB PDF)

Figure S3 **Number of serotypes required to account for 60%, 70%, and 80% of IPD in young children by region.** Error bars indicate the 95% CIs. *Upper bound of 95% CI is >21 serotypes.
(0.03 MB PDF)

Figure S4 **Map of the number of isolates from each country with pneumococcal serotype data included in the analysis.** Grey areas are countries without any serotyped isolates in the analysis.
(0.05 MB PDF)

Figure S5 **Cumulative proportion of IPD in young children due to the 21 most common or important serotypes in each region.** Cumulative proportion of IPD including (red line with square marker) and excluding the single country in the region contributing the greatest number of isolates to the analysis (blue line with circle marker) for (A) Africa and (B) Asia.
(0.02 MB PDF)

Figure S6 **Proportion of IPD in young children due to the serotypes in the existing PCV7 by region.** Assumes serotype 6A/B cross-protection. PCV7 serotypes include: 4, 6B, 9V, 14, 28C, 19F, 23F. PCV10 adds serotypes: 1, 5, and 7F. PCV13 adds serotypes: 3, 6A, and 19A.
(0.05 MB PDF)

Figure S7 **Proportion of IPD in young children due to the serotypes in the existing PCV10 by region.** Assumes serotype 6A/B cross-protection. PCV10 serotypes include: 4, 6B, 9V, 14, 28C, 19F, 23F, 1, 5, and 7F.
(0.05 MB PDF)

Figure S8 **Proportion of IPD in young children due to the serotypes in the existing PCV13 by region.** PCV13 serotypes include: 4, 6B, 9V, 14, 28C, 19F, 23F, 1, 5, 7F, 3, 6A, and 19A.
(0.05 MB PDF)

Table S1 **Search strategy to identify potentially relevant studies in the published literature.**
(0.04 MB DOC)

Table S2 **Steps in selection of serotypes modeled based on a preliminary analysis restricted to studies with all isolates serotyped.** Step 1: Perform meta-analysis of serotype prevalence for all 90 serotypes among studies reporting serotype data (i.e., excluding studies reporting only serogroup data). Step 2: Compare the rank and relative serotype prevalence of serotypes globally and regionally to identify the most common serotypes (as seen below). N, serotype not included in the 23-valent polysaccharide pneumococcal vaccine; N America, North America; PS-23, serotype included in the 23-valent polysaccharide pneumococcal vaccine; RelProp, relative proportion; ST, serotype; Y, included in the 23-valent polysaccharide pneumococcal vaccine.
(0.09 MB DOC)

Table S3 **Characteristics of studies included in the analysis (** ***n*** **= 169).** GAVI Alliance–eligible countries are indicated by (G). Studies reporting only serogroup data are indicated by (X). Unpublished data indicated by PC (personal communication) and supplemental data (†).
(0.34 MB DOC)

Table S4 **Comparison of the ICC for the serotype-specific proportions of IPD among young children reported across studies in each geographic region.** Only the 20 most common serotypes occurring globally are presented. Higher ICC values indicate a higher degree of correlation. Serotypes not observed in a region are indicated by “-.”
(0.05 MB DOC)

Table S5 **Proportion (%) of IPD in young children due to each serotype by region.** 95% CI [27].
(0.07 MB DOC)

Table S6 **Regional incidence of pneumococcal disease (per 100,000) in young children attributed to each serotype.** Pneumococcal disease incidence was estimated by applying the proportion of pneumonia cases caused by SP derived from efficacy estimates from vaccine trials to WHO country-specific estimates of all-cause pneumonia cases then multiplied by country estimates of populations younger than 5 y of age (for more details on pneumococcal disease incidence estimates see [2]). LB, lower bound of uncertainty estimate; UB, upper bound of uncertainty estimate; SSI, serotype-specific incidence (per 100,000 children <5 y of age).
(0.07 MB DOC)

Table S7 **Pneumococcal disease cases in young children attributed to each serotype by region.** Pneumococcal disease cases were estimated by applying the proportion of pneumonia cases caused by SP derived from efficacy estimates from vaccine trials to WHO country-specific estimates of all-cause pneumonia cases among children younger than 5 y of age (for more details on pneumococcal disease cases estimates see [2]). LB, lower bound of uncertainty estimate; UB, upper bound of uncertainty estimate.
(0.08 MB DOC)

Table S8 **Pneumococcal disease mortality (per 100,000) in young children due to each serotype globally, and by region.** Pneumococcal disease mortality was estimated by applying the proportion of pneumonia cases caused by SP derived from efficacy estimates from vaccine trials to WHO country-specific estimates of all-cause pneumonia deaths then multiplied by country estimates of populations younger than 5 y of age (for more details on pneumococcal disease mortality estimates see [2]). LB, lower bound of uncertainty estimate; UB, upper bound of uncertainty estimate; SSM, serotype-specific mortality (per 100,000 children <5 y of age).
(0.08 MB DOC)

Table S9 **Pneumococcal disease deaths in young children attributed to each serotype by region.** Pneumococcal disease deaths were estimated by applying the proportion of pneumonia caused by SP derived from efficacy estimates from vaccine trials to WHO country-specific estimates of all-cause pneumonia deaths among children younger than 5 y of age (for more details on pneumococcal disease deaths estimates see [2]). LB, lower bound of uncertainty estimate; UB, upper bound of uncertainty estimate.
(0.08 MB DOC)

Table S10 **The number of studies in our analysis with serotype data from children age ≥60 mo, by region.**
(0.03 MB DOC)

Text S1 **PRISMA checklist.**
(0.07 MB DOC)

## Abbreviations

AMC: Advance Market Commitment
CI: confidence interval
GAVI: Global Alliance for Vaccines and Immunisation
GBD: global burden of disease
ICC: intraclass correlation
IPD: invasive pneumococcal disease
LAC: Latin America and Caribbean
NA: North America
PCV: pneumococcal conjugate vaccine
PD: pneumococcal disease
SP: *Streptococcus pneumoniae*
TPP: target product profile
WHO: World Health Organization

## References

[1] World Health Organization (2008). The global burden of disease: 2004 update.

[2] O'Brien KL, Wolfson LJ, Watt JP, Henkle E, Deloria-Knoll M (2009). The global burden of disease due to *Streptococcus pneumoniae* in children less than 5 years of age.. Lancet.

[3] Hausdorff WP, Bryant J, Paradiso PR, Siber GR (2000). Which pneumococcal serogroups cause the most invasive disease: implications for conjugate vaccine formulation and use, part I.. Clin Infect Dis.

[4] Cherian T (2007). WHO expert consultation on serotype composition of pneumococcal conjugate vaccines for use in resource-poor developing countries, 26–27 October 2006, Geneva.. Vaccine.

[5] World Health Organization (2009). Global literature review of *Haemophilus influenzae* type b and *Streptococcus pneumoniae* invasive disease among children less than five years of age 1980–2005.

[6] DerSimonian R, Laird N (1986). Meta-analysis in clinical trials.. Controlled Clinical Trials.

[7] United Nations Population Division World Population Prospects: The 2004 Revision Analytical Report.. http://www.un.org/esa/population/publications/WPP2004/WPP2004_Vol3_Final/Preface_TOC_ExpNotes.pdf.

[8] Fleiss JL (1986). The design and analysis of clinical experiments.

[9] Efron B, Tibshirani RJ (1993). An introduction to the bootstrap.

[10] Whitney CG, Farley MM, Hadler J, Harrison LH, Bennett NM (2003). Decline in invasive pneumococcal disease after the introduction of protein-polysaccharide conjugate vaccine.. N Engl J Med.

[11] Whitney CG, Pilishvili T, Farley MM, Schaffner W, Craig AS (2006). Effectiveness of seven-valent pneumococcal conjugate vaccine against invasive pneumococcal disease: a matched case-control study.. Lancet.

[12] Eskola J, Takala AK, Kilpi TM, Lankinen KS, Kayhty H (1998). Clinical evaluation of new pneumococcal vaccines: the Finnish approach.. Dev Biol Stand.

[13] Park IH, Pritchard DG, Cartee R, Brandao A, Brandileone MC (2007). Discovery of a new capsular serotype (6C) within serogroup 6 of *Streptococcus pneumoniae*.. J Clin Microbiol.

[14] Park IH, Park S, Hollingshead SK, Nahm MH (2007). Genetic basis for the new pneumococcal serotype, 6C.. Infect Immun.

[15] Dagan R (2009). Serotype replacement in perspective.. Vaccine.

[16] Constenla DGE, Pio de la Hoz F, O'Loughlin R, Sinha A, Valencia JE (2009). The burden of pneumococcal disease and cost-effectiveness of a pneumococcal vaccine in Latin America and the Caribbean: a review of the evidence and a preliminary economic analysis.

[17] Yaro S, Lourd M, Traoré Y, Njanpop-Lafourcade BM, Sawadogo A (2006). Epidemiological and molecular characteristics of a highly lethal pneumococcal meningitis epidemic in Burkina Faso.. Clin Infect Dis.

[18] Fenoll A, Granizo JJ, Aguilar L, Giménez MJ, Aragoneses-Fenoll L (2009). Temporal trends of invasive Streptococcus pneumoniae serotypes and antimicrobial resistance patterns in Spain from 1979 to 2007.. J Clin Microbiol.

[19] Lagos R, Muñoz A, San Martin O, Maldonado A, Hormazabal JC (2008). Age- and serotype-specific pediatric invasive pneumococcal disease: insights from systematic surveillance in Santiago, Chile, 1994–2007.. J Infect Dis.

[20] Knoll MD, Moisi JC, Muhib FB, Wonodi CB, Lee EH (2009). Standardizing surveillance of pneumococcal disease.. Clin Infect Dis.

[21] Levine OS, Cherian T, Hajjeh R, Knoll MD (2009). Progress and future challenges in coordinated surveillance and detection of pneumococcal and Hib disease in developing countries.. Clin Infect Dis.

[22] Rodenburg GD, de Greeff SC, Jansen AG, de Melker HE, Schouls LM (2010). Effects of pneumococcal conjugate vaccine 2 years after its introduction, the Netherlands.. Emerg Infect Dis.

[23] Imöhl M, Reinert RR, van der Linden M (2010). Temporal variations among invasive pneumococcal disease serotypes in children and adults in Germany (1992–2008).. Int J Microbiol.

[24] Winther TN, Kristensen TD, Kaltoft MS, Konradsen HB, Knudsen JD (2009). Invasive pneumococcal disease in Danish children, 1996–2007, prior to the introduction of heptavalent pneumococcal conjugate vaccine.. Acta Paediatr.

[25] World Health Organization (2003). Pneumococcal vaccines.. Wkly Epidemiol Rec.

[26] United Nations (2001). General Assembly, 56th session. Road map towards the implementation of the United Nations millennium declaration: report of the Secretary-General New York.

[27] PneumoADIP (2008). Pneumococcal Regional Serotype Distribution for Pneumococcal TPP.. http://www.vaccineamc.org/files/TPP_Codebook.pdf.

