# Supplementary material for: Systematic Evaluation of Serotypes Causing Invasive Pneumococcal Disease among Children Under Five: The Pneumococcal Global Serotype Project
Source: PLoS Med. 2010 Oct 5;7(10):e1000348. doi: 10.1371/journal.pmed.1000348 (PMC2950132; doi:10.1371/journal.pmed.1000348)
Supplement: Figure S1 — Flow chart of study identification. Details of excluded studies can be found in the text. *Other sources of data include unpublished data received from contacted researchers. (0.07 MB DOC) [file pmed.1000348.s001.doc]

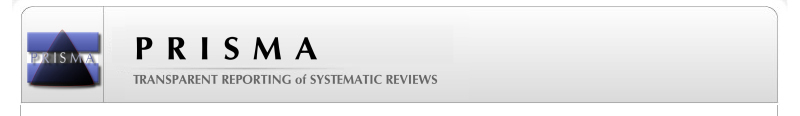
**PRISMA 2009 Flow Diagram**

**Screening**

**Included**

**Eligibility**

**Identification**

Records identified through database searching
(n = 1232)

Additional records identified through other sources*
(n = 60)

Records after duplicates removed
(n = 1248)

Records screened*
(n = 1248)

Records excluded
(n = 630)

Full-text articles assessed for eligibility*
(n = 618)

Full-text articles excluded, with reasons
(n = 449)

Studies included in quantitative synthesis (meta-analysis)
(n = 169)

***Screening/Eligibility Criteria**:

1) ≥ 20 serotyped isolates of *Streptococcus pneumoniae* from a sterile site in children age 0-83 months of age in North America and Europe and age 0-215 months in studies from all other regions.

2) isolates of SP obtained in studies conducted after year 1979

3) studies with at least 12 months of continuous data collection

4) pre-pneumococcal conjugate vaccine introduction
