# Supplementary figures and images for: Systematic Evaluation of Serotypes Causing Invasive Pneumococcal Disease among Children Under Five: The Pneumococcal Global Serotype Project
Source: PLoS Med. 2010 Oct 5;7(10):e1000348. doi: 10.1371/journal.pmed.1000348 (PMC2950132; doi:10.1371/journal.pmed.1000348)

**A**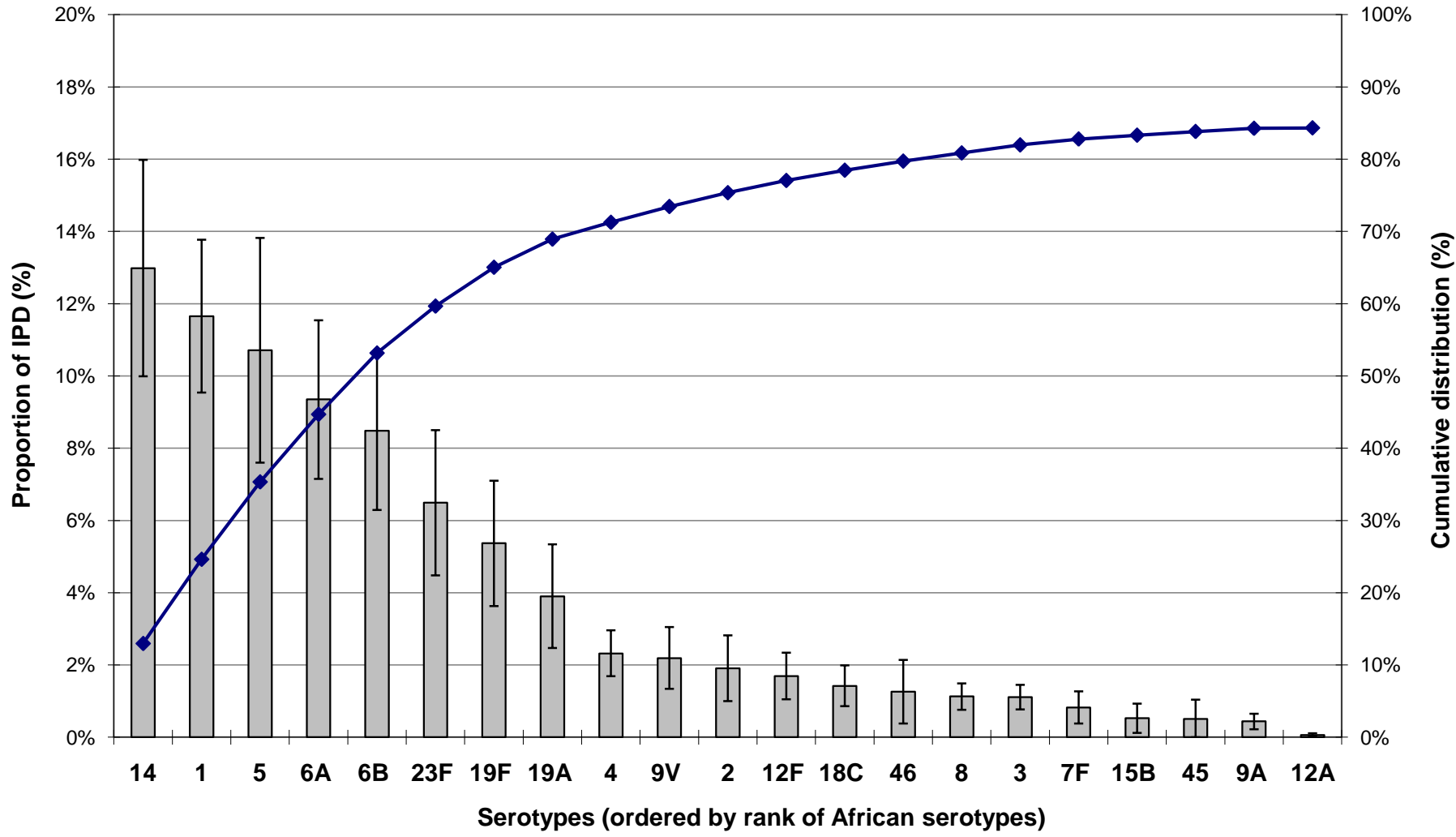

**B**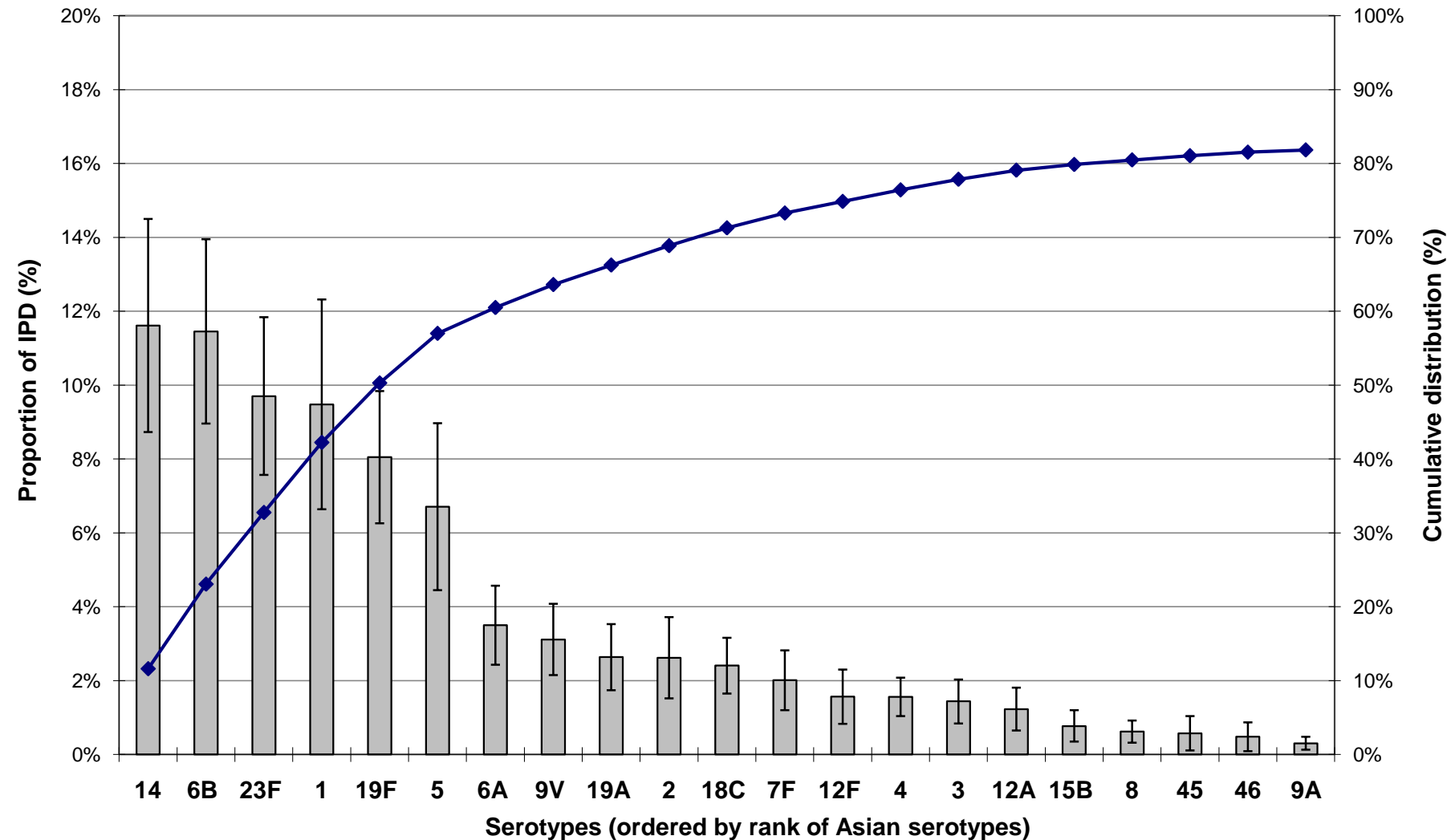

**C**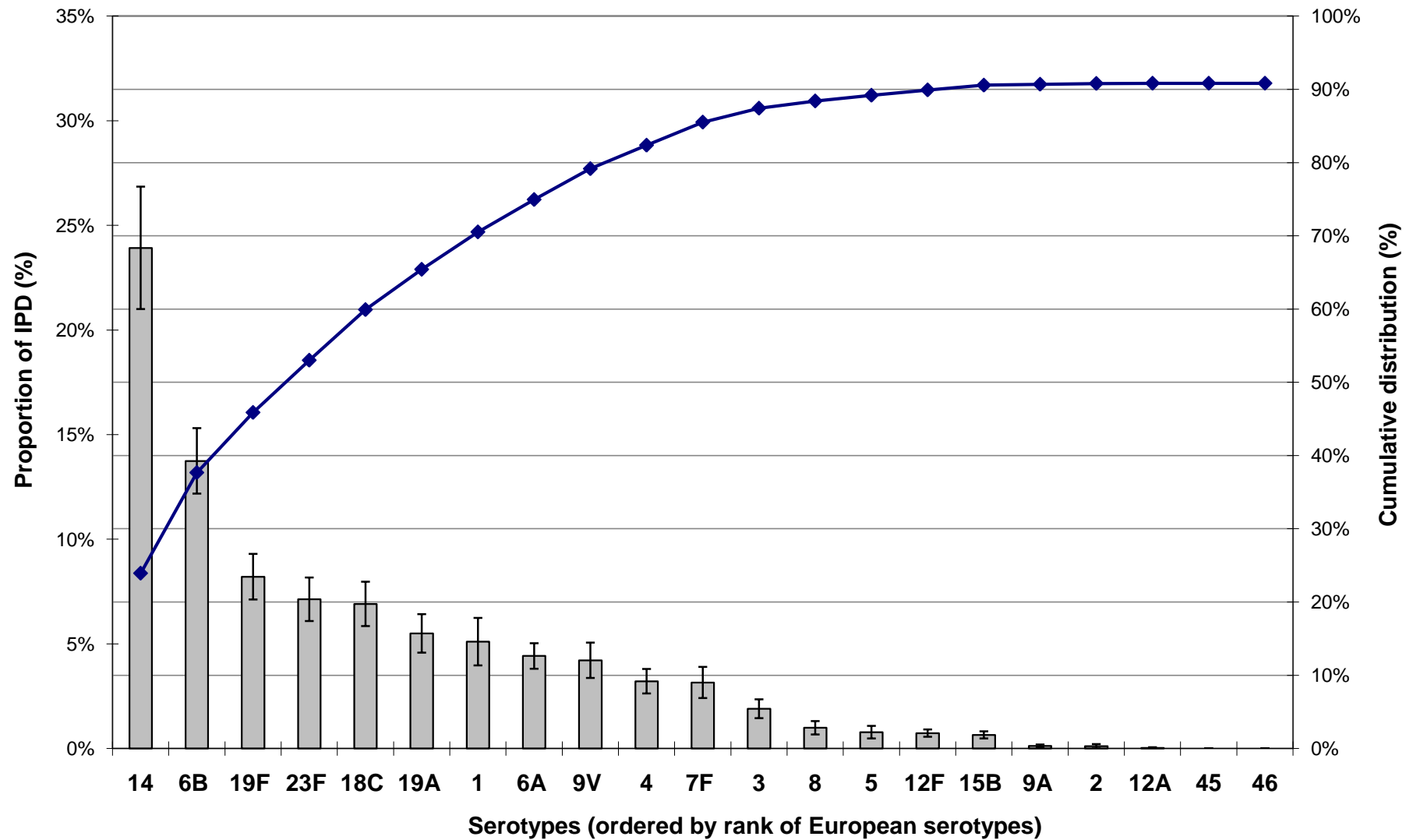

**D**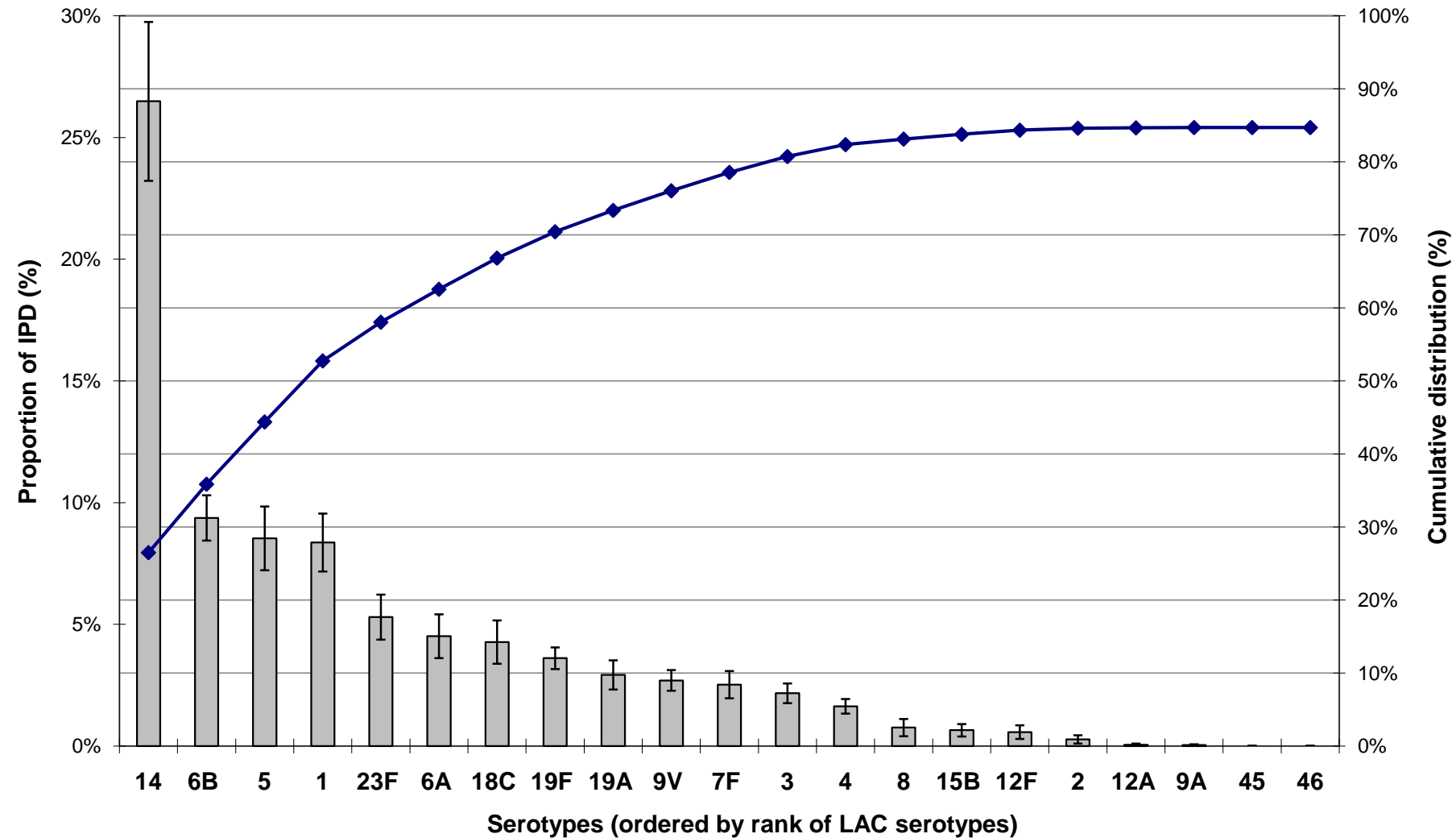

**E**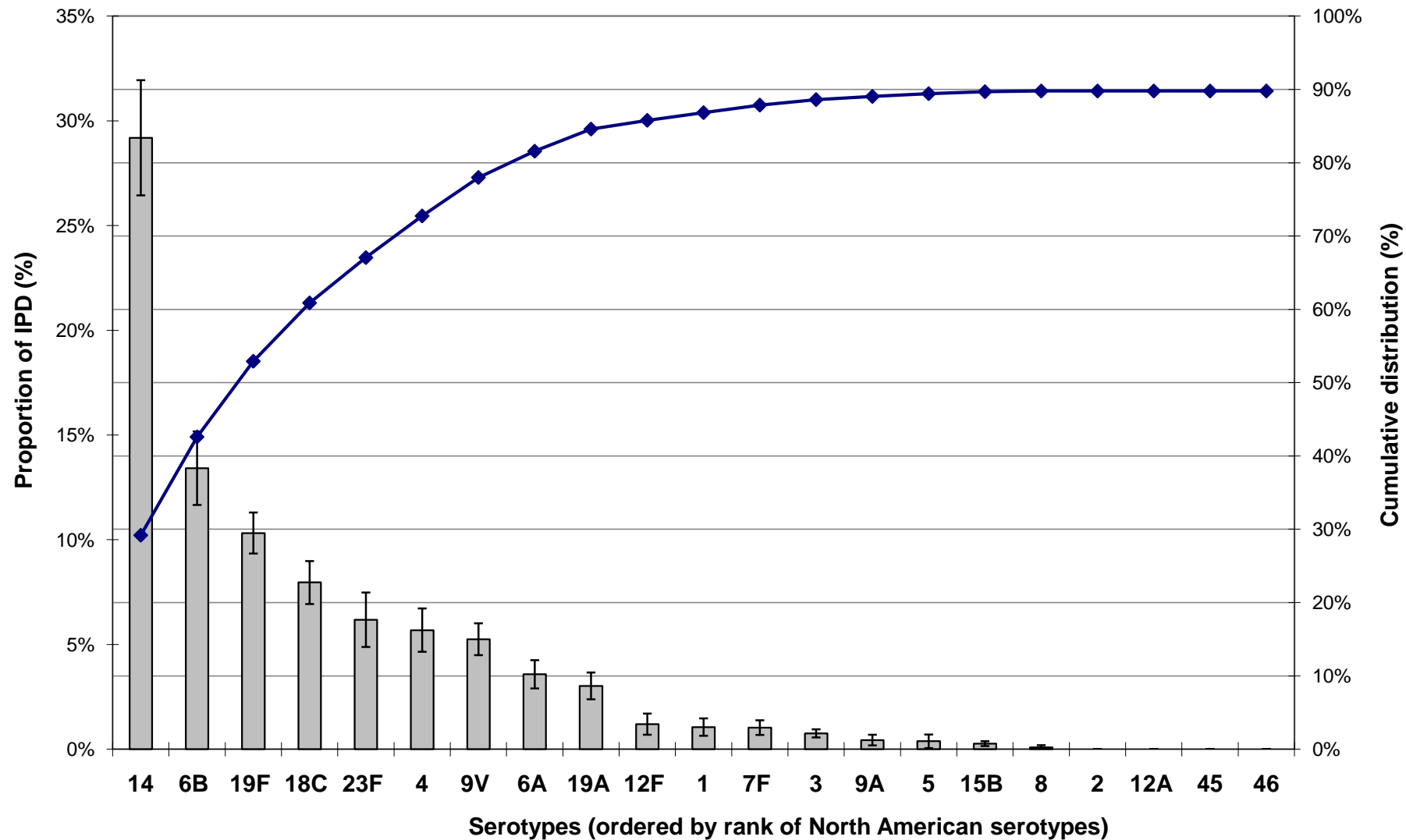

**F**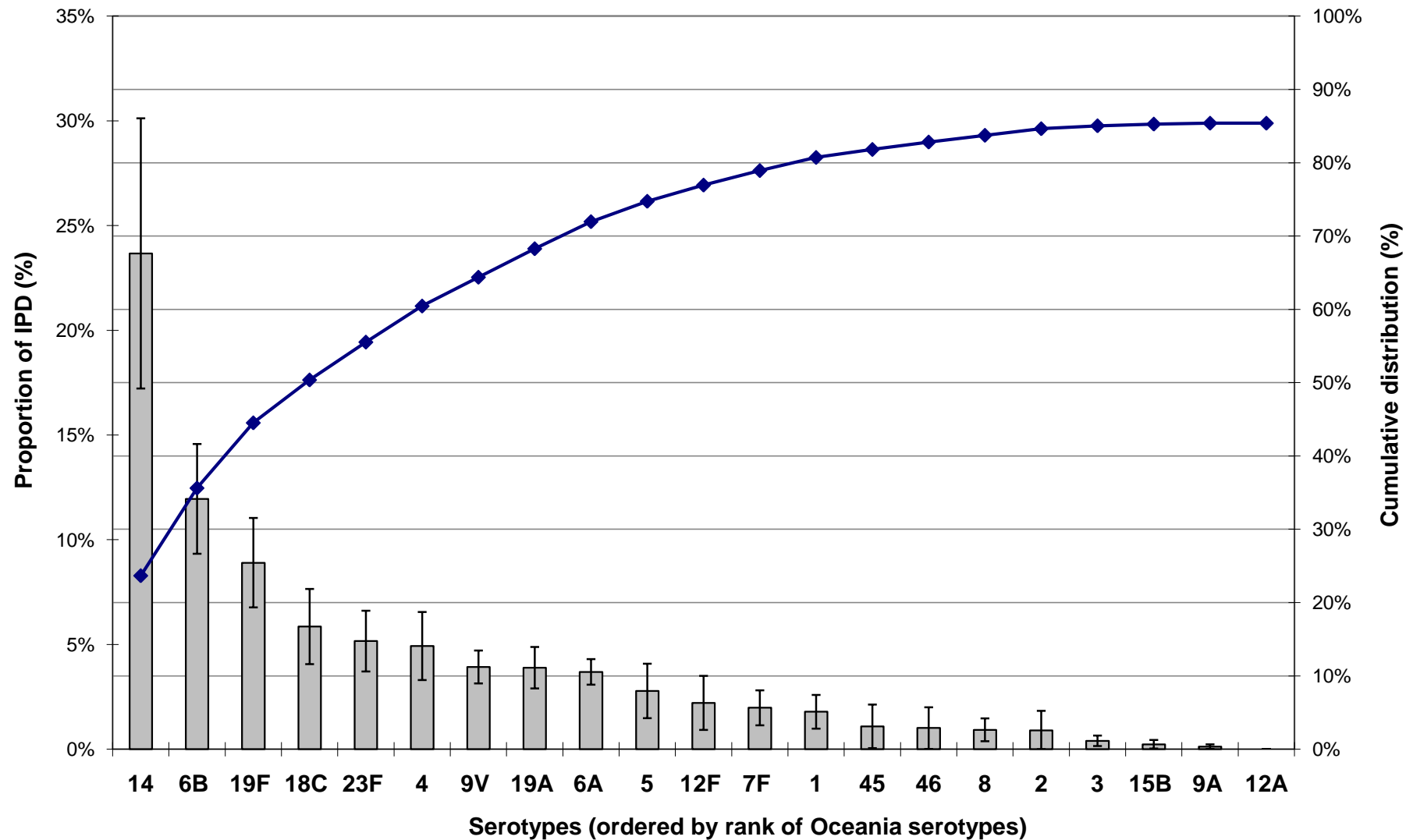

Supplement: Figure S2 — Proportion of IPD in young children due to the 21 most common or important serotypes by region. (A) Africa, (B) Asia, (C) Europe, (D) LAC, (E) NA, (F) Oceania. Error bars indicate the 95% CIs. Line indicates the cumulative proportion across serotypes [27]. (0.51 MB PDF) [file pmed.1000348.s002.pdf]

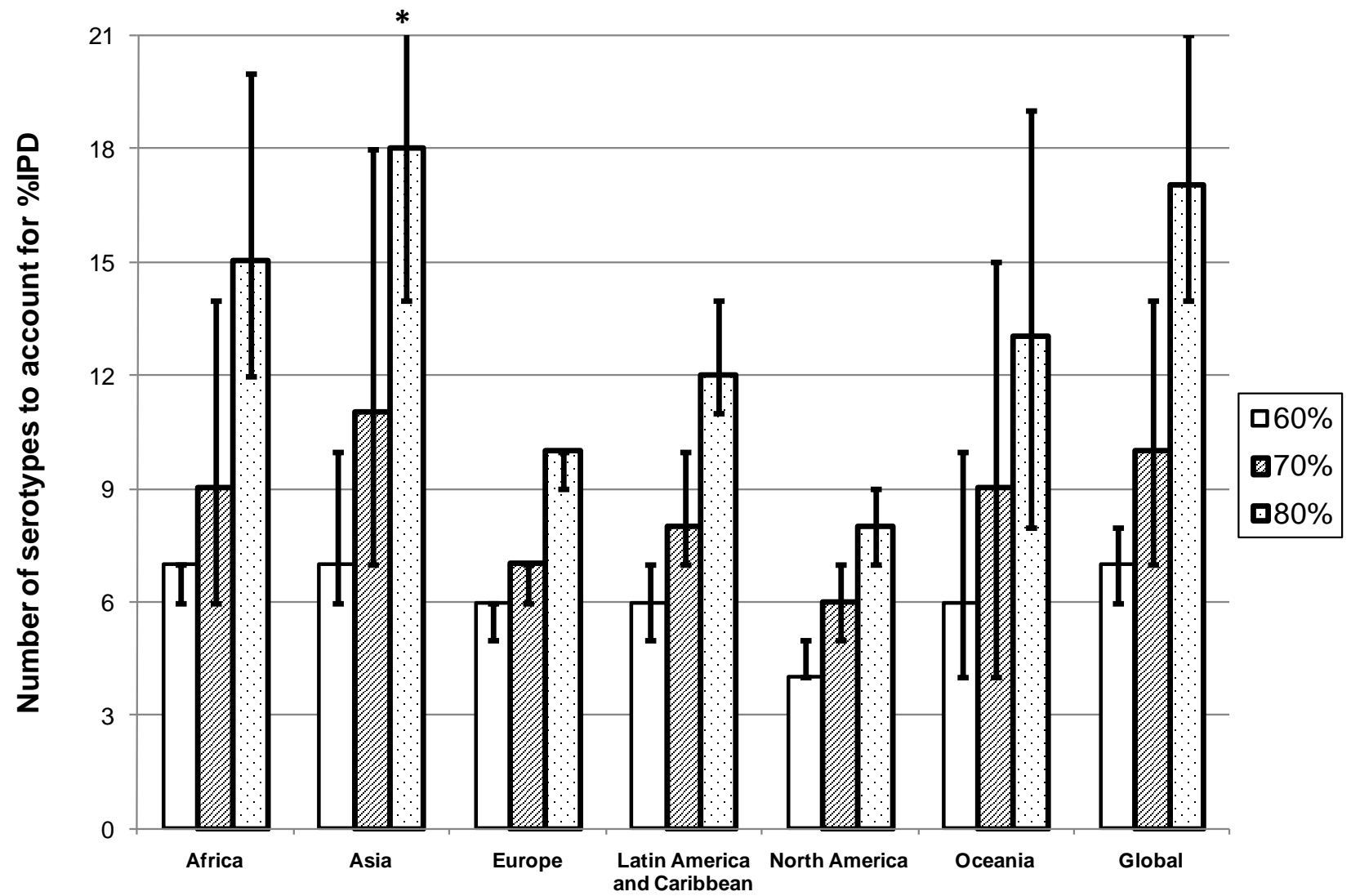

Supplement: Figure S3 — Number of serotypes required to account for 60%, 70%, and 80% of IPD in young children by region. Error bars indicate the 95% CIs. *Upper bound of 95% CI is >21 serotypes. (0.03 MB PDF) [file pmed.1000348.s003.pdf]

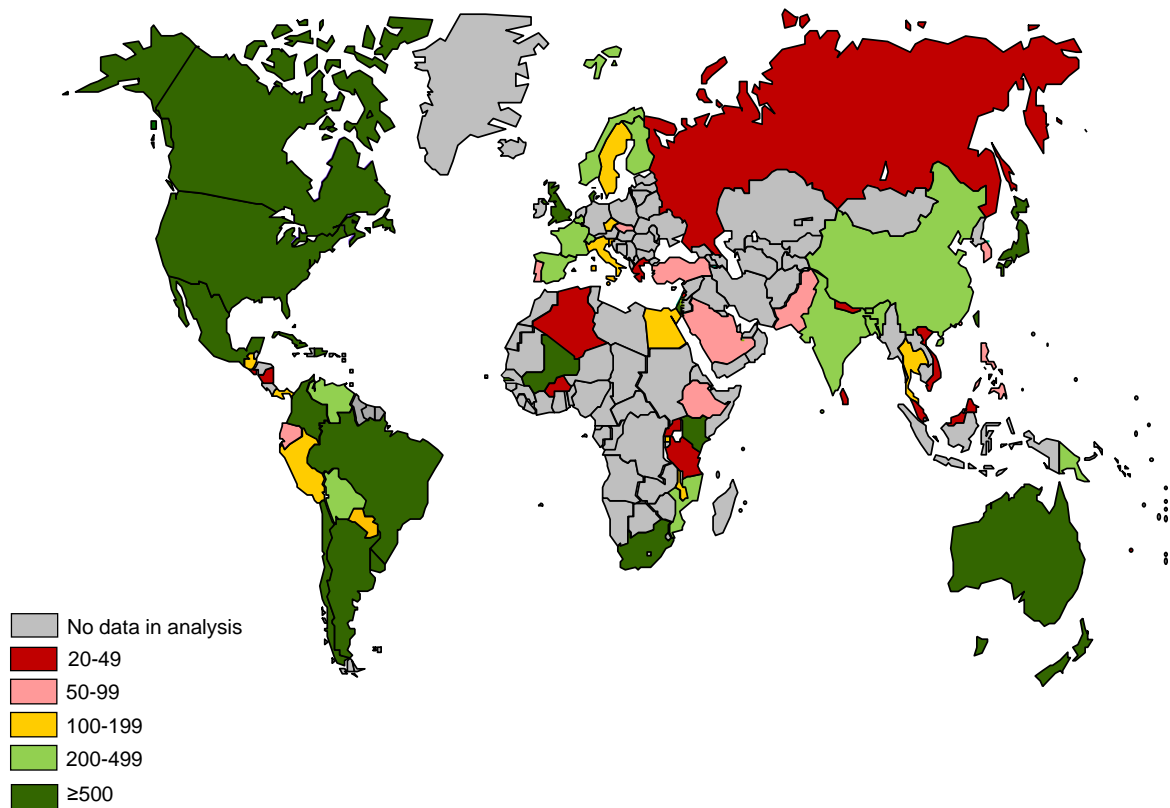

Supplement: Figure S4 — Map of the number of isolates from each country with pneumococcal serotype data included in the analysis. Grey areas are countries without any serotyped isolates in the analysis. (0.05 MB PDF) [file pmed.1000348.s004.pdf]

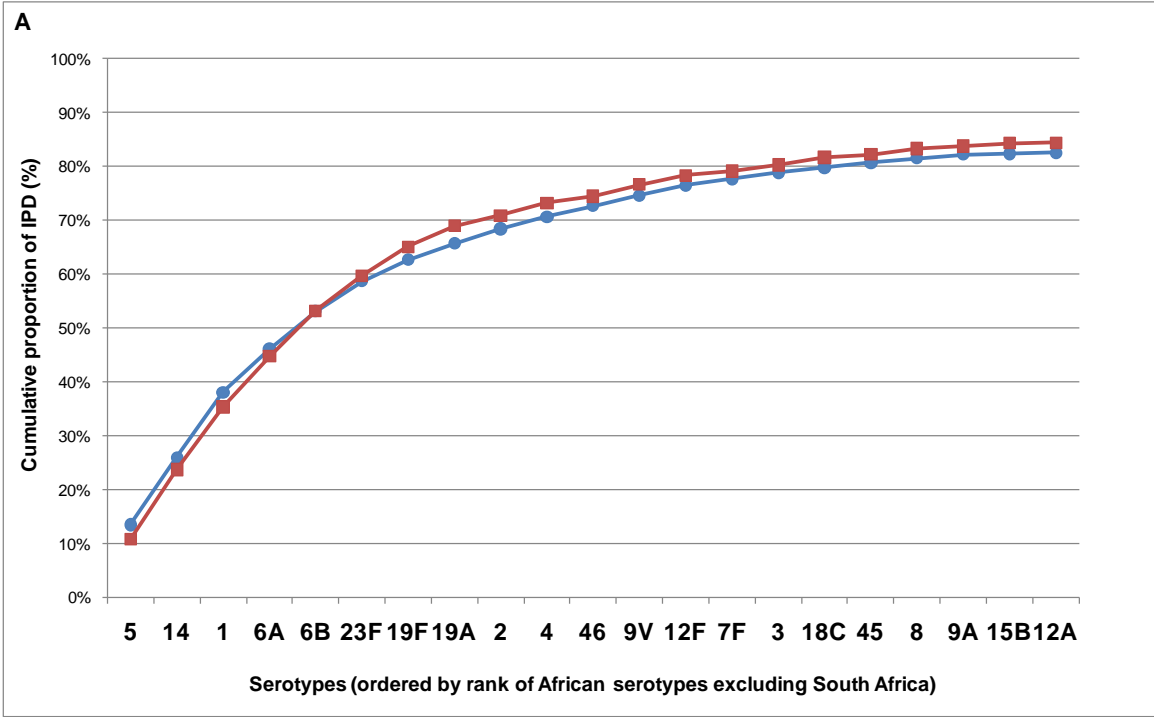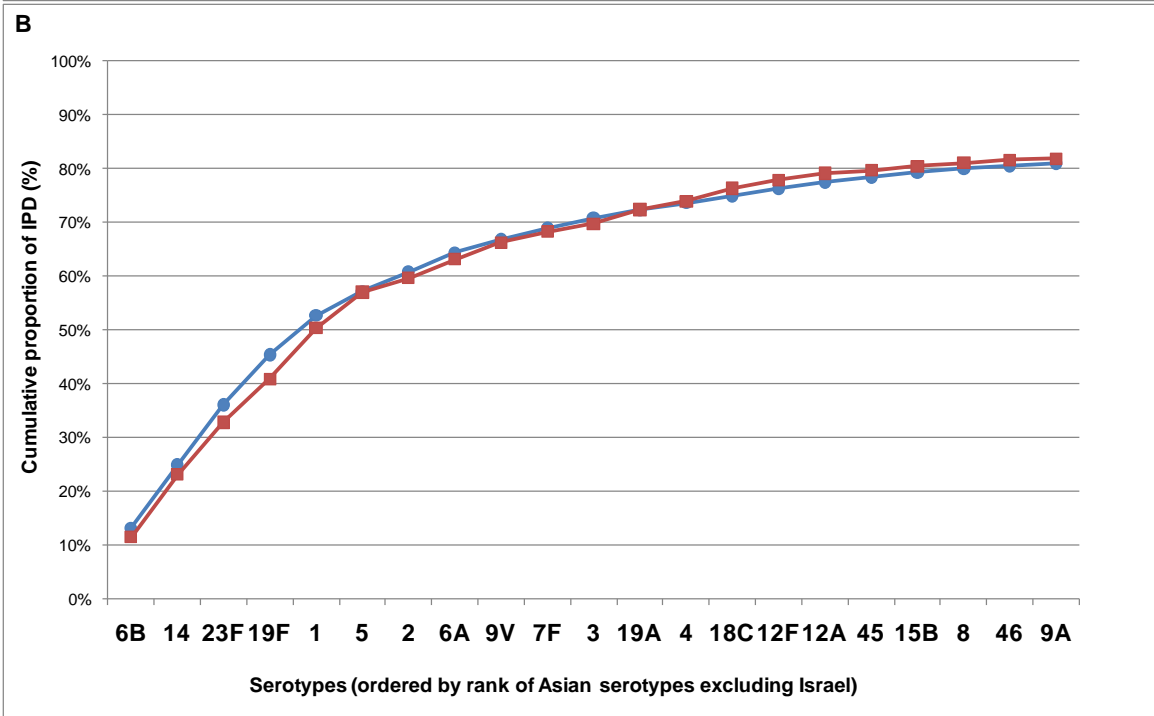

Supplement: Figure S5 — Cumulative proportion of IPD in young children due to the 21 most common or important serotypes in each region. Cumulative proportion of IPD including (red line with square marker) and excluding the single country in the region contributing the greatest number of isolates to the analysis (blue line with circle marker) for (A) Africa and (B) Asia. (0.02 MB PDF) [file pmed.1000348.s005.pdf]

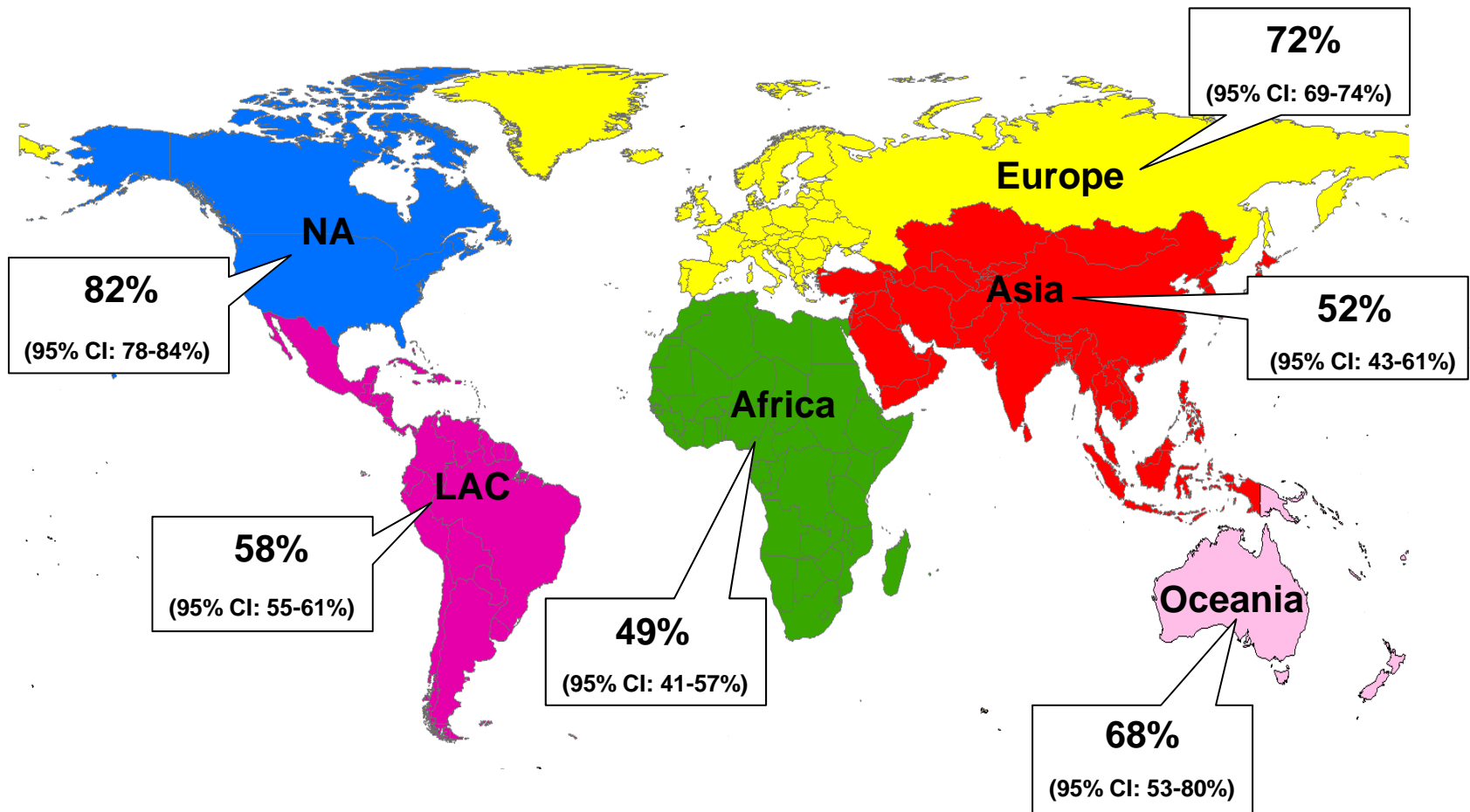

Supplement: Figure S6 — Proportion of IPD in young children due to the serotypes in the existing PCV7 by region. Assumes serotype 6A/B cross-protection. PCV7 serotypes include: 4, 6B, 9V, 14, 28C, 19F, 23F. PCV10 adds serotypes: 1, 5, and 7F. PCV13 adds serotypes: 3, 6A, and 19A. (0.05 MB PDF) [file pmed.1000348.s006.pdf]

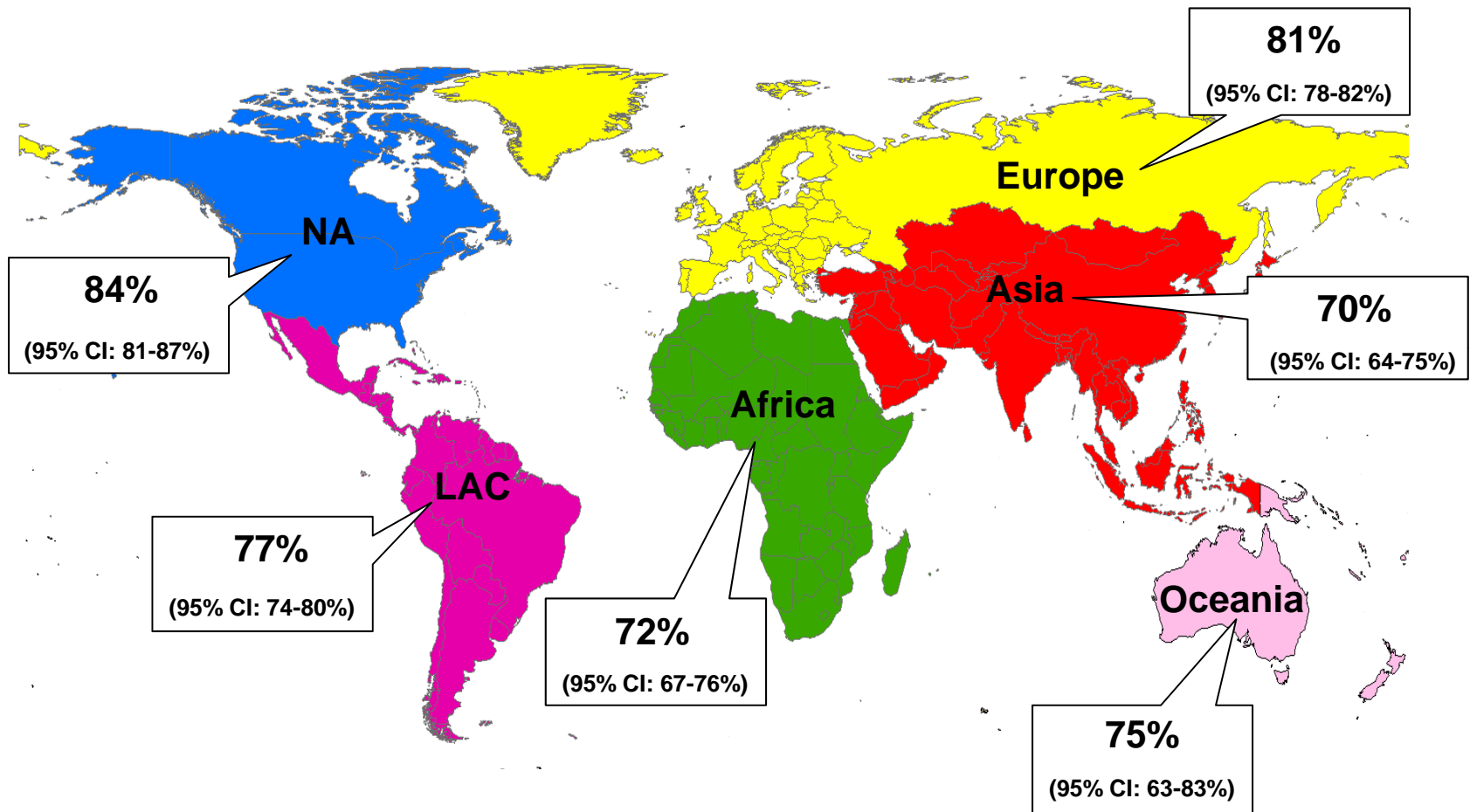

Supplement: Figure S7 — Proportion of IPD in young children due to the serotypes in the existing PCV10 by region. Assumes serotype 6A/B cross-protection. PCV10 serotypes include: 4, 6B, 9V, 14, 28C, 19F, 23F, 1, 5, and 7F. (0.05 MB PDF) [file pmed.1000348.s007.pdf]

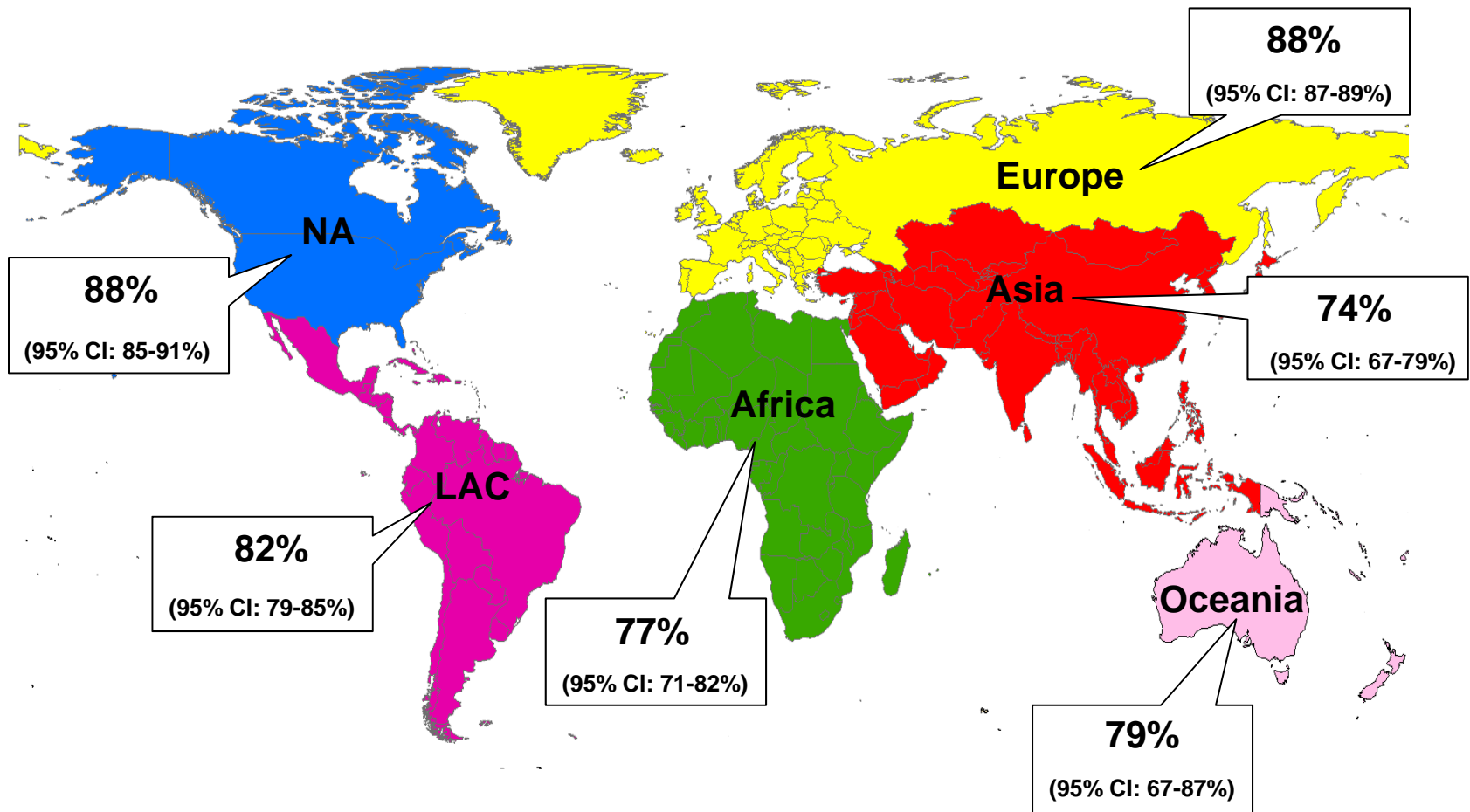

Supplement: Figure S8 — Proportion of IPD in young children due to the serotypes in the existing PCV13 by region. PCV13 serotypes include: 4, 6B, 9V, 14, 28C, 19F, 23F, 1, 5, 7F, 3, 6A, and 19A. (0.05 MB PDF) [file pmed.1000348.s008.pdf]
