# Supplementary material for: Systematic Evaluation of Serotypes Causing Invasive Pneumococcal Disease among Children Under Five: The Pneumococcal Global Serotype Project
Source: PLoS Med. 2010 Oct 5;7(10):e1000348. doi: 10.1371/journal.pmed.1000348 (PMC2950132; doi:10.1371/journal.pmed.1000348)
Supplement: Table S1 — Search strategy to identify potentially relevant studies in the published literature. (0.04 MB DOC) [file pmed.1000348.s009.doc]

**Table S1.** Search strategy to identify potentially relevant studies in the published literature.

| 1. **Search Terms** |
| --- |
| **MeSH Terms for pneumococcus:** Streptococcus pneumoniae[mh] OR pneumonia, pneumococcal[mh] OR meningitis, pneumococcal[mh] OR pneumococcal vaccines[mh] OR pneumococcal infections |
| **A.2. Key Word Search for pneumococcus:** (Streptococcus()pneumoniae or diplococcus()pneumoniae or pneumococcus or s()pneumoniae or pneumococci or pneumococc?()vaccine? or pneumococc?()polysaccharide or pneumococcal()conjugate or pneumovax()23 or prevnar or pneumococcal()heptavalent()conjugate or pnu()imune()23 or pnu()imune()vaccine? or 7()valent()pncompc()vaccine? or seven()valent()pneumococcal()ps or meningococcal()ompc()conjugate()vaccine? or pncrm7 or mnc()crm197 or mncc or pneumovax or streptococcus()pneumoniae()meningitis or pneumococcal()meningitis or pneumococcal()pneumonia or pneumococcal()bacteremia or pneumococcal()bacteraemia or invasive()pneumococcal or pneumoccal()mortality) |
| 1. **Limiter Terms** |
| **B.1. Child:** neonate? OR neonatal OR newborn? OR new()born? OR infant? OR infancy OR baby OR babies OR toddler? OR preschool OR child? OR juvenile? OR girl? OR boy?OR young? OR youth? OR teenager? OR adolescen? OR pediatric? OR paediatric? |
| **B.2. Human*** |
| **B.3. Serotype* OR Serogroup*** |
| **B.4. Geographic areas:** mauritius or seychelles or cape()verde or namibia or south()africa or eritrea or kenya or sudan or uganda or gambia or ghana or senegal or togo or comoros or djibouti or madagascar or zimbabwe or congo or gabon or sao()tome or principe or botswana or lesotho or swaziland or ethiopia or cameroon or central()african()republic or chad or benin or burkina()faso or cote(2w)ivoire or ivory()coast or guinea or guinea()bissau or mali or mauritania or niger or nigeria or burundi or malawi or mozambique or rwanda or somalia or tanzania or zambia or angola or congo or liberia or sierra()leone or brunei or malaysia or singapore or thailand or japan or korea or sri()lanka or bahrain or cyprus or georgia or israel or kuwait or oman or qatar or saudi()arabia or syria or syrian()arab()republic or united()arab()emirates or indonesia or philippines or vietnam or viet()nam or china or iran or kyrgyzstan or uzbekistan or armenia or jordan or lebanon or turkey or cambodia or timor()leste or east()timor or myanmar or burma or mongolia or bangladesh or bhutan or india or kazakhstan or maldives or nepal or pakistan or tajikistan or turkmenistan or azerbaijan or iraq or yemen or lao or laos or afghanistan or taiwan or macao or borneo or antigua or barbuda or bahamas or barbados or cuba or dominica or grenada or jamaica or saint()kitts or nevis or saint()lucia or saint()vincent or grenadines or trinidad or tobago or costa()rica or mexico or panama or argentina or chile or colombia or ecuador or uruguay or venezuela or dominican()republic or belize or el()salvador or guatemala or honduras or nicaragua or brazil or paraguay or peru or suriname or haiti or bolivia or guyana or puerto()rico or virgin()islands or french()guiana |
