# Supplementary material for: Systematic Evaluation of Serotypes Causing Invasive Pneumococcal Disease among Children Under Five: The Pneumococcal Global Serotype Project
Source: PLoS Med. 2010 Oct 5;7(10):e1000348. doi: 10.1371/journal.pmed.1000348 (PMC2950132; doi:10.1371/journal.pmed.1000348)
Supplement: Table S2 — Steps in selection of serotypes modeled based on a preliminary analysis restricted to studies with all isolates serotyped. Step 1: Perform meta-analysis of serotype prevalence for all 90 serotypes among studies reporting serotype data (i.e., excluding studies reporting only serogroup data). Step 2: Compare the rank and relative serotype prevalence of serotypes globally and regionally to identify the most common serotypes (as seen below). N, serotype not included in the 23-valent polysaccharide pneumococcal vaccine; N America, North America; PS-23, serotype included in the 23-valent polysaccharide pneumococcal vaccine; RelProp, relative proportion; ST, serotype; Y, included in the 23-valent polysaccharide pneumococcal vaccine. (0.09 MB DOC) [file pmed.1000348.s010.doc]

**Table S2.** Steps in selection of serotypes modeled based on a preliminary analysis restricted to studies with all isolates serotyped. LAC = Latin America and the Caribbean region; N = Not included in the Serotype included in the 23-valent polysaccharide pneumococcal vaccine; N America = North America; PS-23 = Serotype included in the 23-valent polysaccharide pneumococcal vaccine; RelProp = relative proportion; ST = serotype; Y = Included in the 23-valent polysaccharide pneumococcal vaccine.

Step 1: Perform meta-analysis of serotype prevalence for all 90 serotypes among studies reporting serotype data (i.e. excluding studies reporting only serogroup data).

Step 2: Compare the rank and relative serotype prevalence of serotypes globally and regionally to identify the most common serotypes (as seen below).

|  | **Number** | **Global Adjusted** | | | **In PS-23** | **Rank in regional ST distribution** | | | | | |
| --- | --- | --- | --- | --- | --- | --- | --- | --- | --- | --- | --- |
|  |  | **Rank** | **Serotype** | **RelProp** |  | **Africa** | **Asia** | **Europe** | **LAC** | **N America** | **Oceania** |
| **Top 23 from global adjusted** | 1 | 1 | 14 | 14.10% | Y | 1 | 1 | 1 | 1 | 1 | 1 |
|  | 2 | 2 | 1 | 10.25% | Y | 2 | 4 | 5 | 4 | 10 | 11 |
|  | 3 | 3 | 6B | 9.57% | Y | 5 | 2 | 2 | 2 | 2 | 2 |
|  | 4 | 4 | 5 | 8.47% | Y | 3 | 6 | 14 | 3 | 20 | 10 |
|  | 5 | 5 | 23F | 7.86% | Y | 6 | 3 | 4 | 5 | 6 | 6 |
|  | 6 | 6 | 19F | 7.18% | Y | 7 | 5 | 3 | 8 | 3 | 3 |
|  | 7 | 7 | 6A | 5.54% | N | 4 | 7 | 8 | 6 | 8 | 9 |
|  | 8 | 8 | 19A | 3.48% | Y | 8 | 9 | 7 | 9 | 9 | 8 |
|  | 9 | 9 | 9V | 3.03% | Y | 9 | 8 | 9 | 10 | 7 | 7 |
|  | 10 | 10 | 18C | 2.21% | Y | 14 | 11 | 6 | 7 | 4 | 4 |
|  | 11 | 11 | 2 | 2.20% | Y | 10 | 10 | 39 | 26 | 0 | 16 |
|  | 12 | 12 | 4 | 1.79% | Y | 11 | 12 | 10 | 13 | 5 | 5 |
|  | 13 | 13 | 12F | 1.42% | Y | 12 | 14 | 16 | 17 | 11 | 12 |
|  | 14 | 14 | 7F | 1.33% | Y | 20 | 13 | 11 | 11 | 14 | 21 |
|  | 15 | 15 | 3 | 1.28% | Y | 15 | 15 | 12 | 12 | 13 | 18 |
|  | 16 | 16 | 12A | 0.95% | N | 17 | 16 | 40 | 53 | 0 | 0 |
|  | 17 | 17 | 15B | 0.84% | Y | 27 | 17 | 20 | 16 | 22 | 28 |
|  | 18 | 18 | 8 | 0.77% | Y | 19 | 19 | 13 | 15 | 37 | 13 |
|  | 19 | 19 | 46 | 0.73% | N | 13 | 32 | 0 | 0 | 0 | 15 |
|  | 20 | 20 | 45 | 0.61% | N | 22 | 28 | 0 | 0 | 0 | 14 |
|  | 21 | 21 | 15A | 0.57% | N | 33 | 20 | 35 | 44 | 32 | 0 |
|  | 22 | 22 | 24A | 0.57% | N | 18 | 41 | 0 | 0 | 0 | 0 |
|  | 23 | 23 | 7B | 0.56% | N | 16 | 46 | 48 | 48 | 0 | 0 |
| **Additional PS-23 STs not in top 23 global adjusted** | 24 | 27 | 20 | 0.49% | Y | 30 | 30 | 31 | 27 | 42 | 0 |
|  | 25 | 30 | 9N | 0.44% | Y | 28 | 47 | 18 | 21 | 16 | 17 |
|  | 26 | 41 | 10A | 0.37% | Y | 42 | 40 | 24 | 14 | 24 | 26 |
|  | 27 | 47 | 22F | 0.28% | Y | 23 | 0 | 15 | 29 | 15 | 25 |
|  | 28 | 56 | 17F | 0.25% | Y | 56 | 55 | 33 | 20 | 39 | 30 |
|  | 29 | 59 | 11A | 0.24% | Y | 36 | 63 | 22 | 23 | 25 | 27 |
|  | 30 | 63 | 33F | 0.19% | Y | 51 | 60 | 17 | 33 | 18 | 24 |
| **Top 20 regional STs not in top 23 global or in additional PS-23** | 31 | 24 | 10F | 0.54% | N | 46 | 18 | 61 | 41 | 0 | 0 |
|  | 32 | 25 | 9A | 0.50% | N | 24 | 34 | 46 | 52 | 12 | 0 |
|  | 33 | 28 | 24F | 0.47% | N | 26 | 44 | 19 | 22 | 33 | 0 |
|  | 34 | 36 | 19B | 0.41% | N | 60 | 27 | 44 | 19 | 41 | 0 |
|  | 35 | 37 | 16F | 0.40% | N | 31 | 52 | 27 | 18 | 28 | 33 |
|  | 36 | 38 | 18B | 0.40% | N | 57 | 26 | 34 | 46 | 21 | 20 |
|  | 37 | 42 | 15C | 0.36% | N | 54 | 31 | 23 | 24 | 17 | 22 |
|  | 38 | 62 | 18A | 0.19% | N | 49 | 58 | 47 | 37 | 40 | 19 |
