# Supplementary material for: Systematic Evaluation of Serotypes Causing Invasive Pneumococcal Disease among Children Under Five: The Pneumococcal Global Serotype Project
Source: PLoS Med. 2010 Oct 5;7(10):e1000348. doi: 10.1371/journal.pmed.1000348 (PMC2950132; doi:10.1371/journal.pmed.1000348)
Supplement: Table S3 — Characteristics of studies included in the analysis ( n = 169). GAVI Alliance–eligible countries are indicated by (G). Studies reporting only serogroup data are indicated by (X). Unpublished data indicated by PC (personal communication) and supplemental data (†). (0.34 MB DOC) [file pmed.1000348.s011.doc]

**Table S3.** Characteristics of studies included in the analysis (N=169). GAVI Alliance-eligible countries are indicated by (G). Studies reporting only serogroup data are indicated by (X). Unpublished data indicated by PC (Personal Communication) and supplemental data (†)

|  | Country | Reference | | | Study years | | | | | | Serogroup Only Data | | | | | | | | Total No. isolates | | | | | | | |  |
| --- | --- | --- | --- | --- | --- | --- | --- | --- | --- | --- | --- | --- | --- | --- | --- | --- | --- | --- | --- | --- | --- | --- | --- | --- | --- | --- | --- |
| AFRICA (N=22 studies) | |  | | |  | | | | | |  | | | | | | | | | | | |  | | |  | |
| 1. | Algeria | [Ramdani, 2003] [1†] | | | 1996-2000 | | | | | |  | | | | | | | | | | | | 45 | | |  | |
| 2. | Burkina Faso (G) | [Gessner, 2009] [2†] | | | 2002-2005 | | | | | |  | | | | | | | | | | | | 22 | | |  | |
| 3. | Egypt | [Wasfy, 2005] [3†] | | | 1998-2003 | | | | | |  | | | | | | | | | | | | 113 | | |  | |
| 4. | Ethiopia (G) | [Muhe, 1999] [4] | | | 1993-1995 | | | | | |  | | | | | | | | | | | | 46 | | |  | |
| 5. | Kenya (G) | [Scott, 1998] [5†] | | | 1994-2007 | | | | | |  | | | | | | | | | | | | 595 | | |  | |
| 6. | Kenya (G) | [[Mudhune, 2009] [6†] | | | 2004-2007 | | | | | |  | | | | | | | | | | | | 46 | | |  | |
| 7. | Malawi (G) | [Gordon, 2003] [7] | | | 1996-1998 | | | | | | X | | | | | | | | | | | | 122 | | |  | |
| 8. | Mali (G) | [Mariko, 2005] [8] | | | 2003-2004 | | | | | |  | | | | | | | | | | | | 54 | | |  | |
| 9. | Mali (G) | [Campbell, 2004] [9†] | | | 2002-2007 | | | | | |  | | | | | | | | | | | | 570 | | |  | |
| 10. | Mozambique (G) | [Roca, 2009] [10†] | | | 2001-2007 | | | | | |  | | | | | | | | | | | | 259 | | |  | |
| 11. | Rwanda (G) | [Bogaerts, 1993] [11] | | | 1984-1990 | | | | | | X | | | | | | | | | | | | 130 | | |  | |
| 12. | South Africa | [Friedland, 1992] [12] | | | 1989-1991 | | | | | | X | | | | | | | | | | | | 181 | | |  | |
| 13. | South Africa | [Crewe-Brown, 1997] [13] | | | 1993-1995 | | | | | | X | | | | | | | | | | | | 98 | | |  | |
| 14. | South Africa | [Klugman, 2003] [14] | | | 1998-2001 | | | | | |  | | | | | | | | | | | | 66 | | |  | |
| 15. | South Africa | [Wolter, 2008] [15†] | | | 2000-2006 | | | | | |  | | | | | | | | | | | | 8221 | | |  | |
| 16. | Tanzania (G) | [Mudhune, 2009] [6†] | | | 2006-2007 | | | | | |  | | | | | | | | | | | | 27 | | |  | |
| 17. | The Gambia (G) | [Usen, 1998] [16] | | | 1993-1995 | | | | | | X | | | | | | | | | | | | 105 | | |  | |
| 18. | The Gambia (G) | [O’Dempsey, 1996] [17] | | | 1989-1991 | | | | | | X | | | | | | | | | | | | 60 | | |  | |
| 19. | The Gambia (G) | [Adegbola, 2006] [18†] | | | 1996-2003 | | | | | |  | | | | | | | | | | | | 212 | | |  | |
| 20. | The Gambia (G) | [Gordon, 2002] [19] | | | 2000-2003 | | | | | |  | | | | | | | | | | | | 116 | | |  | |
| 21. | The Gambia (G) | [Adegbola, 1994] [20] | | | 1990-1992 | | | | | | X | | | | | | | | | | | | 46 | | |  | |
| 22. | Uganda (G) | [Mudhune, 2009] [6†] | | | 2004-2007 | | | | | |  | | | | | | | | | | | | 47 | | |  | |
| ASIA (N=33 studies) | |  | | |  | | | | | |  | | | | | | | | | | | |  | | |  | |
| 23. | Bangladesh (G) | [Saha, 1997] [21] | | | 1992-1995 | | | | | |  | | | | | | | | | | | | 151 | | |  | |
| 24. | Bangladesh (G) | [Saha, 2005] [22] | | | 2001-2004 | | | | | | X | | | | | | | | | | | | 63 | | |  | |
| 25. | Bangladesh (G) | [Saha, 2005] [22†] | | | 2002-2004 | | | | | |  | | | | | | | | | | | | 46 | | |  | |
| 26. | Bangladesh (G) | [Saha, 2009] [23†] | | | 2004-2007 | | | | | |  | | | | | | | | | | | | 151 | | |  | |
| 27. | Bangladesh (G) | [Saha, 2009] [23†] | | | 2004-2007 | | | | | |  | | | | | | | | | | | | 51 | | |  | |
| 28. | China | [Shen, 1989] [24] | | | 1982-1985 | | | | | | X | | | | | | | | | | | | 236 | | |  | |
| 29. | China, Hong Kong | [Ho, 2004] [25] | | | 1995-2001 | | | | | |  | | | | | | | | | | | | 88 | | |  | |
| 30. | China, Taiwan | [Siu, 2002] [26] | | | 1998-1999 | | | | | | X | | | | | | | | | | | | 75 | | |  | |
| 31. | China, Taiwan | [Lauderdale, 2006] [27] | | | 2002-2003 | | | | | |  | | | | | | | | | | | | 76 | | |  | |
| 32. | China, Taiwan | [Chen, 2006] [28] | | | 2002-2003 | | | | | |  | | | | | | | | | | | | 147 | | |  | |
| 33. | China, Taiwan | [Lin, 2006] [29] | | | 1999-2004 | | | | | |  | | | | | | | | | | | | 286 | | |  | |
| 34. | India (G) | [Lakshmy, 1997] [30] | | | 1988-1992 | | | | | | X | | | | | | | | | | | | 115 | | |  | |
| 35. | India (G) | [IBIS, 1999] [31] | | | 1993-1997 | | | | | | X | | | | | | | | | | | | 101 | | |  | |
| 36. | Israel | [Dagan, 1992] [32] | | | 1988-1990 | | | | | |  | | | | | | | | | | | | 213 | | |  | |
| 37. | Israel | [Rahav, 1997] [33] | | | 1987-1992 | | | | | |  | | | | | | | | | | | | 119 | | |  | |
| 38. | Israel | [Shouval, 2006] [34] | | | 2000-2004 | | | | | |  | | | | | | | | | | | | 184 | | |  | |
| 39. | Israel | [Ron Dagan, PC] | | | 1999-2006 | | | | | |  | | | | | | | | | | | | 1146 | | |  | |
| 40. | Israel | [Fraser, 2001] [35] | | | 1989-1998 | | | | | |  | | | | | | | | | | | | 463 | | |  | |
| 41. | Japan | [Ubukata, 2004] [36] | | | 1999-2002 | | | | | |  | | | | | | | | | | | | 138 | | |  | |
| 42. | Japan | [Sakata, 2006] [37] | | | 1998-2005 | | | | | |  | | | | | | | | | | | | 39 | | |  | |
| 43. | Japan | [Chiba, 2010] [38†] | | | 2002-2004 | | | | | |  | | | | | | | | | | | | 231 | | |  | |
| 44. | Lebanon | [Ghassan Dbaibo, PC] | | | 2004-2007 | | | | | |  | | | | | | | | | | | | 24 | | |  | |
| 45. | Malaysia | [Rohani, 1999] [39] | | | 1994-1995 | | | | | |  | | | | | | | | | | | | 20 | | |  | |
| 46. | Nepal (G) | [Shah, 2009; Williams, 2009] [40†,41†] | | | 2004-2006 | | | | | |  | | | | | | | | | | | | 49 | | |  | |
| 47. | Pakistan (G) | [Mastro, 1991] [42] | | | 1986-1988 | | | | | |  | | | | | | | | | | | | 87 | | |  | |
| 48. | Philippines | [Capeding, 1994] [43] | | | 1988-1997 | | | | | | X | | | | | | | | | | | | 65 | | |  | |
| 49. | Saudi Arabia | [Al Mazrou, 2005] [44] | | | 2000-2001 | | | | | | X | | | | | | | | | | | | 51 | | |  | |
| 50. | South Korea | [Choi, 1998] [45] | | | 1985-1996 | | | | | |  | | | | | | | | | | | | 56 | | |  | |
| 51. | Sri Lanka (G) | [Batuwanthudawe, 2009] [46†] | | | 2005-2007 | | | | | |  | | | | | | | | | | | | 21 | | |  | |
| 52. | Thailand | [Levine, 2006] [47†] | | | 2005-2007 | | | | | |  | | | | | | | | | | | | 58 | | |  | |
| 53. | Thailand | [Phongsamart, 2007] [48] | | | 2000-2005 | | | | | |  | | | | | | | | | | | | 115 | | |  | |
| 54. | Turkey | [Yalcin, 2006] [49] | | | 2001-2004 | | | | | |  | | | | | | | | | | | | 60 | | |  | |
| 55. | Vietnam (G) | [Parry, 2002] [50] | | | 1993-2002 | | | | | |  | | | | | | | | | | | | 27 | | |  | |
| EUROPE (N=39 studies) | |  | | |  | | | | | |  | | | | | | | | | | | |  | | |  | |
| 56. | Austria | [Rendi-Wagner, 2004] [51] | | | 2001-2003 | | | | | |  | | | | | | | | | | | | 56 | | |  | |
| 57. | Belgium | [Vergison, 2006] [52] | | | 2002-2003 | | | | | |  | | | | | | | | | | | | 280 | | |  | |
| 58. | Belgium | [Verhaegen, 2003] [53†] | | | 1997-2000 | | | | | | X | | | | | | | | | | | | 86 | | |  | |
| 59. | Belgium | [De Schutter, 2006] [54] | | | 1988-2002 | | | | | |  | | | | | | | | | | | | 55 | | |  | |
| 60. | Czech Republic | [Prymula, 2004] [55] | | | 1999-2000 | | | | | |  | | | | | | | | | | | | 108 | | |  | |
| 61. | Denmark | [Kaltoft, 2000] [56] | | | 1981-1999 | | | | | |  | | | | | | | | | | | | 1123 | | |  | |
| 62. | Finland | [Eskola, 1992] [57] | | | 1985-1989 | | | | | | X | | | | | | | | | | | | 235 | | |  | |
| 63. | Finland | [Hanage, 2005] [58] | | | 1995-1999 | | | | | |  | | | | | | | | | | | | 224 | | |  | |
| 64. | France | [Decousser, 2004] [59] | | | 1997-2002 | | | | | |  | | | | | | | | | | | | 41 | | |  | |
| 65. | France | [Doit, 2002] [60†] | | | 1992-1997 | | | | | |  | | | | | | | | | | | | 309 | | |  | |
| 66. | France | [Bekri, 2007] [61] | | | 2002-2004 | | | | | |  | | | | | | | | | | | | 25 | | |  | |
| 67. | Greece | [Levidiotou, 2006] [62] | | | 2000-2004 | | | | | |  | | | | | | | | | | | | 46 | | |  | |
| 68. | Italy | [Tarallo, 2006] [63] | | | 2002-2003 | | | | | |  | | | | | | | | | | | | 42 | | |  | |
| 69. | Italy | [Pantosti, 2000] [64] | | | 1997-1999 | | | | | |  | | | | | | | | | | | | 25 | | |  | |
| 70. | Italy | [Pantosti, 2003] [65] | | | 1997-2000 | | | | | |  | | | | | | | | | | | | 51 | | |  | |
| 71. | Norway | [Magnus, 2005] [66] | | | 1985-1990 | | | | | |  | | | | | | | | | | | | 24 | | |  | |
| 72. | Norway | [Pedersen, 2004] [67] | | | 1995-2001 | | | | | |  | | | | | | | | | | | | 325 | | |  | |
| 73. | Portugal | [Serrano, 2004] [68†] | | | 1999-2002 | | | | | |  | | | | | | | | | | | | 80 | | |  | |
| 74. | Russia | [Vishniakova, 1981] [69] | | | 1978-1980 | | | | | | X | | | | | | | | | | | | 37 | | |  | |
| 75. | Slovenia | [Paragi, 2003] [70] | | | 1993-2001 | | | | | |  | | | | | | | | | | | | 195 | | |  | |
| 76. | Spain | [Nava, 1994] [71] | | | 1987-1992 | | | | | | X | | | | | | | | | | | | 42 | | |  | |
| 77. | Spain | [Calbo, 2006] [72] | | | 1999-2004 | | | | | |  | | | | | | | | | | | | 57 | | |  | |
| 78. | Spain | [Latorre, 2004] [73] | | | 1989-2000 | | | | | |  | | | | | | | | | | | | 92 | | |  | |
| 79. | Spain | [Pineda, 2002] [74] | | | 1990-2002 | | | | | | X | | | | | | | | | | | | 25 | | |  | |
| 80. | Spain | [Barricarte, 2007] [75] | | | 2001-2004 | | | | | |  | | | | | | | | | | | | 152 | | |  | |
| 81. | Spain | [Aristegui, 2007] [76] | | | 1998-2003 | | | | | | X | | | | | | | | | | | | 59 | | |  | |
| 82. | Spain | [Canton, 2003] [77] | | | 1999-2002 | | | | | | X | | | | | | | | | | | | 38 | | |  | |
| 83. | Sweden | [Hedlund, 1995] [78†] | | | 1987-1992 | | | | | |  | | | | | | | | | | | | 130 | | |  | |
| 84. | Sweden | [Ekdahl, 1998] [79†] | | | 1981-1996 | | | | | | X | | | | | | | | | | | | 21 | | |  | |
| 85. | Sweden | [Berg, 2006] [80] | | | | | 1998-2001 | | |  | | | | | | | | | | | | | 26 | | |  | |
| 86. | Switzerland | [Kronenberg, 2006] [81] | | | | | 2002-2004 | | |  | | | | | | | | | | | | | 204 | | |  | |
| 87. | United Kingdom | [Urwin, 1996] [82] | | | | | 1990-1993 | | |  | | | | | | | | | | | | | 28 | | |  | |
| 88. | United Kingdom | [Colman, 1998] [83] | | | | | 1982-1990 | | | X | | | | | | | | | | | | | 576 | | |  | |
| 89. | United Kingdom | [Brueggemann, 2003] [84] | | | | | 1995-2001 | | |  | | | | | | | | | | | | | 150 | | |  | |
| 90. | United Kingdom | [Kyaw, 2000] [85] | | | | | 1988-1999 | | | X | | | | | | | | | | | | | 246 | | |  | |
| 91. | United Kingdom | [Miller, 2000] [86] | | | | | 1996-1998 | | | X | | | | | | | | | | | | | 1112 | | |  | |
| 92. | United Kingdom | [Shackley, 2000] [87] | | | | | 1991-1996 | | | X | | | | | | | | | | | | | 80 | | |  | |
| 93. | United Kingdom | [Johnson, 2007] [88†] | | | | | 1998-2005 | | |  | | | | | | | | | | | | | 3657 | | |  | |
| 94. | United Kingdom | [Clarke, 2006] [89] | | | 2000-2004 | | | | | |  | | | | | | | | | | | | 217 | | |  | |
| Latin America and the Caribbean (N=42 studies) | | | | |  | | | | | |  | | | | | | | | | | | |  | | |  | |
| 95. | Argentina | [Kertesz, 1998] [90] | | | | 1993-1996 | | | | | | |  | | | | | | | | | 374 | | | |  | |
| 96. | Argentina | [Tregnaghi, 2006] [91] | | | | 1999-2002 | | | | | | |  | | | | | | | | | 179 | | | |  | |
| 97. | Argentina | [PAHO, 2007] [92] | | | | 2000-2005 | | | | | | |  | | | | | | | | | 936 | | | |  | |
| 98. | Bolivia (G) | [PAHO, 2007] [92] | | | | 2000-2005 | | | | | | |  | | | | | | | | | 149 | | | |  | |
| 99. | Brazil | [Brandileone, 1995] [98] | | | | 1977-1993 | | | | | | |  | | | | | | | | | 645 | | | |  | |
| 100. | Brazil | [Brandileone, 1997] [94] | | | | 1993-1996 | | | | | | |  | | | | | | | | | 360 | | | |  | |
| 101. | Brazil | [Kertesz, 1998] [90] | | | | | | | 1993-1996 | | |  | | | | | | | | 323 | | | | | | |  |
| 102. | Brazil | [Nascimento-Carvalho, 2003] [95†] | | | | | | | 1997-2002 | | |  | | | | | | | | 53 | | | | | | |  |
| 103. | Brazil | [Mantese, 2003] [96†] | | | | | | | 1999-2003 | | |  | | | | | | | | 76 | | | | | | |  |
| 104. | Brazil | [Reis, 2002] [97] | | | | | | | 1995-1999 | | |  | | | | | | | | 145 | | | | | | |  |
| 105. | Brazil | [Brandileone, 2003] [98†] | | | | | | | 1997-2000 | | |  | | | | | | | | 2341 | | | | | | |  |
| 106. | Brazil | [Magalhäes, 2003] [99] | | | | | | | 2000-2001 | | |  | | | | | | | | 31 | | | | | | |  |
| 107. | Brazil | [Vieira, 2007] [100] | | | | | | | 1995-2004 | | |  | | | | | | | | 173 | | | | | | |  |
| 108. | Brazil | [Taunay, 1990] [101] | | | | | | | 1977-1988 | | |  | | | | | | | | 466 | | | | | | |  |
| 109. | Brazil | [Berezin, 2007] [102] | | | | | | | 1996-2001 | | |  | | | | | | | | 105 | | | | | | |  |
| 110. | Brazil | [PAHO, 2007] [92] | | | | | | | 2000-2005 | | |  | | | | | | | | 1975 | | | | | | |  |
| 111. | Chile | [Levine, 1998] [103] | | | | | | | 1989-1996 | | |  | | | | | | | | 66 | | | | | | |  |
| 112. | Chile | [Kertesz, 1998] [90] | | | | | | | 1993-1996 | | |  | | | | | | | | 173 | | | | | | |  |
| 113. | Chile | [Contreras, 2002] [104] | | | | | | | 1994-1999 | | |  | | | | | | | | 78 | | | | | | |  |
| 114. | Chile | [Inostroza, 2001] [105] | | | | | | | 1994-1999 | | |  | | | | | | | | 69 | | | | | | |  |
| 115. | Chile | [PAHO, 2007] [92] | | | | | | | 2000-2005 | | |  | | | | | | | | 1893 | | | | | | |  |
| 116. | Colombia | [Kertesz, 1998] [90] | | | | | | | 1993-1996 | | |  | | | | | | | | 290 | | | | | | |  |
| 117. | Colombia | [Agudelo, 2006] [106] | | | | | | | 1994-2004 | | |  | | | | | | | | 2231 | | | | | | |  |
| 118. | Colombia | [PAHO, 2007] [92] | | | | | | | 2000-2005 | | |  | | | | | | | | 647 | | | | | | |  |
| 119. | Cuba (G) | [PAHO, 2007] [92] | | | | | | | 2000-2005 | | |  | | | | | | | | 841 | | | | | | |  |
| 120. | Dominican Republic | [Castaneda, 2009] [107†] | | | | | | | 2006-2007 | | |  | | | | | | | | 420 | | | | | | |  |
| 121. | Dominican Republic | [PAHO, 2007] [92] | | | | | | | 2000-2005 | | |  | | | | | | | | 394 | | | | | | |  |
| 122. | Ecuador | [PAHO, 2007] [92] | | | | | | | 2000-2005 | | |  | | | | | | | | 55 | | | | | | |  |
| 123. | El Salvador | [PAHO, 2007] [92] | | | | | | | 2000-2005 | | |  | | | | | | | | 23 | | | | | | |  |
| 124. | Guatemala | [Dueger, 2008] [108†] | | | | | | | 2005-2005 | | |  | | | | | | | | 62 | | | | | | |  |
| 125. | Guatemala | [PAHO, 2007] [92] | | | | | | | 2000-2005 | | | X | | | | | | | | 91 | | | | | | |  |
| 126. | Mexico | [Kertesz, 1998] [90] | | | | | | | 1993-1996 | | |  | | | | | | | | 129 | | | | | | |  |
| 127. | Mexico | [PAHO, 2007] [92] | | | | | | | 2000-2005 | | |  | | | | | | | | 562 | | | | | | |  |
| 128. | Nicaragua (G) | [PAHO, 2007] [92] | | | | | | | 2000-2005 | | |  | | | | | | | | 39 | | | | | | |  |
| 129. | Panama | [PAHO, 2007] [92] | | | | | | | 2000-2005 | | |  | | | | | | | | 101 | | | | | | |  |
| 130. | Paraguay | [PAHO, 2007] [92] | | | | | | | 2000-2005 | | |  | | | | | | | | 478 | | | | | | |  |
| 131. | Peru | [PAHO, 2007] [92] | | | | | | | 2000-2005 | | |  | | | | | | | | 132 | | | | | | |  |
| 132. | Uruguay | [Mogdasy, 1992] [109] | | | | | | | 1987-1989 | | |  | | | | | | | | 48 | | | | | | |  |
| 133. | Uruguay | [Kertesz, 1998] [90] | | | | | | | 1993-1996 | | |  | | | | | | | | 146 | | | | | | |  |
| 134. | Uruguay | [Camou, 2003] [110†] | | | | | | | 1994-2001 | | |  | | | | | | | | 506 | | | | | | |  |
| 135. | Uruguay | [PAHO, 2007] [92] | | | | | | | 2000-2005 | | |  | | | | | | | | 575 | | | | | | |  |
| 136. | Venezuela | [PAHO, 2007] [92] | | | | | | | 2000-2005 | | |  | | | | | | | | 408 | | | | | | |  |
| North America (N=17 studies) | | | | |  | | | | | |  | | | | | | | | | | | |  | | | | |
| 137. | Canada | [Kellner, 1998] [111†] | | | | | | 1995-2001 | | | | | |  | | | | | | | | | | 530 | | | |
| 138. | Canada | [Jette, 2001] [112] | | | | | | 1996-1998 | | | | | |  | | | | | | | | | | 339 | | | |
| 139. | Canada | [Bigham, 2003] [113] | | | | | | 1999-2000 | | | | | |  | | | | | | | | | | 33 | | | |
| 140. | Canada | [Kellner, 2009] [114†] | | | | | | 1998-2006 | | | | | |  | | | | | | | | | | 155 | | | |
| 141. | United States of America | [Butler, 1995] [115] | | | | | | 1978-1994 | | | | | |  | | | | | | | | | | 3570 | | | |
| 142. | United States of America | [Zangwill, 1996] [116] | 1992-1995 | | | | | | | | | | | |  | | | | | | | | | | 61 | | |
| 143. | United States of America | [Butler, 1996] [117] | | 1993-1994 | | | | | | | | | | | |  | | 272 | | | | | | | | | |
| 144. | United States of America | [Shapiro, 1994] [118] | | 1984-1993 | | | | | | | | | | | |  | | 502 | | | | | | | | | |
| 145. | United States of America | [Parkinson, 1994] [119] | | 1986-1990 | | | | | | | | | | | |  | | 210 | | | | | | | | | |
| 146. | United States of America | [O’Brien, 2004] [120†] | | 1991-2000 | | | | | | | | | | | |  | | 246 | | | | | | | | | |
| 147. | United States of America | [Adamkiewicz, 2003][121†] | | 1994-1997 | | | | | | | | | | | |  | | 40 | | | | | | | | | |
| 148. | United States of America | [Kaplan, 2002] [122] | | 1993-1999 | | | | | | | | | | | | X | | 2019 | | | | | | | | | |
| 149. | United States of America | [Alpern, 2001] [123] | | 1993-1996 | | | | | | | | | | | |  | | 88 | | | | | | | | | |
| 150. | United States of America | [Rudolph, 2000] [124] | | 1991-1998 | | | | | | | | | | | |  | | 436 | | | | | | | | | |
| 151. | United States of America | [Lexau, 1999] [125†] | | 1995-1997 | | | | | | | | | | | |  | | 470 | | | | | | | | | |
| 152. | United States of America | [Mufson, 2004] [126†] | | 1978-2003 | | | | | | | | | | | | X | | 117 | | | | | | | | | |
| 153. | United States of America | [Moore, 2007] [127†] | | | | | 1998-1999 | | | | | | | | | |  | | | | 2353 | | | | | | |
| Oceania (N=16 studies) | |  | | | | |  | | | |  | | | | | | | | | | | |  | | | | |
| 154. | Australia | [Gratten, 1998] [128] | | | | | 1990-1997 | | | |  | | | | | | | | | | | | 253 | | | | |
| 155. | Australia | [Gratten, 1996] [129] | | | | | 1989-1994 | | | |  | | | | | | | | | | | | 89 | | | | |
| 156. | Australia | [Trotman, 1995] [130] | | | | | 1992-1993 | | | |  | | | | | | | | | | | | 32 | | | | |
| 157. | Australia | [Krause, 2000] [131†] | | | | | 1994-1998 | | | |  | | | | | | | | | | | | 171 | | | | |
| 158. | Australia | [Fagan, 2001] [132] | | | | | 1992-2000 | | | |  | | | | | | | | | | | | 97 | | | | |
| 159. | Australia | [Liu, 2003] [133] | | | | | 2001-2002 | | | |  | | | | | | | | | | | | 107 | | | | |
| 160. | Australia | [Hanna, 2006] [134] | | | | | 1999-2004 | | | |  | | | | | | | | | | | | 46 | | | | |
| 161. | Australia | [Roche, 2006] [135†] | | | | | 2004-2004 | | | |  | | | | | | | | | | | | 610 | | | | |
| 162. | Australia | [Watson, 2004] [136] | | | | | 2003-2003 | | | |  | | | | | | | | | | | | 637 | | | | |
| 163. | Australia | [Giele, 2007] [137] | | | | | 1996-2005 | | | |  | | | | | | | | | | | | 505 | | | | |
| 164. | New Caledonia | [Michel, 2005] [138†] | | | | | 1999-2001 | | | |  | | | | | | | | | | | | 20 | | | | |
| 165. | New Zealand | [Martin, 2006] [139] | | | | | 1987-1994 | | | | X | | | | | | | | | | | | 623 | | | | |
| 166. | New Zealand | [Voss, 1994] [140] | | | | | 1989-1997 | | | | X | | | | | | | | | | | | 129 | | | | |
| 167. | Papua New Guinea (G) | [Gratten, 2005] [141] | | | | | 1978-1987 | | | | X | | | | | | | | | | | | 155 | | | | |
| 168. | Papua New Guinea (G) | [Barker, 1989] [142] | | | | | 1983-1984 | | | | X | | | | | | | | | | | | 29 | | | | |
| 169. | Papua New Guinea (G) | [Lehmann, 1997] [143] | | | | | 1980-1987 | | | | X | | | | | | | | | | | | 146 | | | | |

**References Corresponding to Table S3**
