# Supplementary material for: Systematic Evaluation of Serotypes Causing Invasive Pneumococcal Disease among Children Under Five: The Pneumococcal Global Serotype Project
Source: PLoS Med. 2010 Oct 5;7(10):e1000348. doi: 10.1371/journal.pmed.1000348 (PMC2950132; doi:10.1371/journal.pmed.1000348)
Supplement: Table S4 — Comparison of the ICC for the serotype-specific proportions of IPD among young children reported across studies in each geographic region. Only the 20 most common serotypes occurring globally are presented. Higher ICC values indicate a higher degree of correlation. Serotypes not observed in a region are indicated by “-.” (0.05 MB DOC) [file pmed.1000348.s012.doc]

**Table S4.** Comparison of the intra-class correlation for the serotype-specific proportions of invasive pneumococcal disease among young children reported across studies in each geographic region. Only serotypes for the 20 most common serotypes occurring globally are presented. Higher values of the intra-class correlation indicate higher degree of correlation. Serotypes not observed in a region are indicated by “-“.

| **Serotype** | **Africa** | **Asia** | **Europe** | **Latin America and Caribbean** | **North America** | **Oceania** |
| --- | --- | --- | --- | --- | --- | --- |
| 1 | 0.095 | 0.139 | 0.201 | 0.126 | 0.560 | 0.839 |
| 2 | 0.127 | 0.660 | 1.000 | 0.920 | - | - |
| 3 | 1.000 | 0.801 | 0.708 | 0.459 | 0.989 | 0.801 |
| 4 | 0.405 | 0.711 | 0.405 | 0.587 | 0.245 | 0.669 |
| 5 | 0.020 | 0.145 | 0.808 | 0.112 | 0.805 | 0.863 |
| 6A | 0.055 | 0.225 | 0.269 | 0.374 | 0.405 | 0.994 |
| 6B | 0.049 | 0.127 | 0.181 | 0.249 | 0.175 | 0.941 |
| 7F | 0.334 | 0.546 | 0.257 | 0.243 | 0.813 | 0.906 |
| 8 | 0.483 | 1.000 | 0.669 | 0.605 | 0.979 | 0.961 |
| 9V | 0.230 | 0.483 | 0.278 | 0.503 | 0.575 | 1.000 |
| 12A | 1.000 | 0.798 | 1.000 | 1.000 | - | - |
| 12F | 0.247 | 0.529 | 0.959 | 0.687 | 0.493 | 0.548 |
| 14 | 0.039 | 0.105 | 0.072 | 0.038 | 0.113 | 0.063 |
| 15A | 0.772 | 1.000 | 1.000 | 1.000 | 1.000 | - |
| 15B | 0.177 | 0.607 | 1.000 | 0.837 | 1.000 | 1.000 |
| 18C | 0.212 | 0.551 | 0.225 | 0.128 | 0.503 | 1.000 |
| 19A | 0.068 | 0.279 | 0.293 | 0.371 | 0.336 | 0.393 |
| 19F | 0.078 | 0.193 | 0.226 | 0.571 | 0.509 | 0.458 |
| 23F | 0.049 | 0.164 | 0.309 | 0.175 | 0.196 | 0.309 |
| 45 | 1.000 | 0.839 | - | - | - | 1.000 |
| Other | 0.038 | 0.124 | 0.255 | 0.033 | 0.045 | 0.152 |
