# Supplementary material for: Systematic Evaluation of Serotypes Causing Invasive Pneumococcal Disease among Children Under Five: The Pneumococcal Global Serotype Project
Source: PLoS Med. 2010 Oct 5;7(10):e1000348. doi: 10.1371/journal.pmed.1000348 (PMC2950132; doi:10.1371/journal.pmed.1000348)
Supplement: Table S5 — Proportion (%) of IPD in young children due to each serotype by region. 95% CI [27]. (0.07 MB DOC) [file pmed.1000348.s013.doc]

**Table S5.** Proportion (%) of invasive pneumococcal disease in young children due to each serotype by region. Source: PneumoADIP. Pneumococcal Regional Serotype Distribution for Pneumococcal TPP (2008). 95% Confidence Interval (CI).

Accessed on October 19, 2009: <http://www.vaccineamc.org/files/TPP_Codebook.pdf>

| **Serotype** | **Africa** | | **Asia** | | **Europe** | | **Latin America and Caribbean** | | **North America** | | **Oceania** | | **Global** | |
| --- | --- | --- | --- | --- | --- | --- | --- | --- | --- | --- | --- | --- | --- | --- |
|  | % | 95% CI | % | 95% CI | % | 95% CI | % | 95% CI | % | 95% CI | % | 95% CI | % | 95% CI |
| 1 | 11.7% | 9.5, 13.8 | 9.5% | 6.6, 12.3 | 5.1% | 4.0, 6.2 | 8.4% | 7.2, 9.6 | 1.1% | 0.6, 1.5 | 1.8% | 1.0, 2.6 | 10.0% | 5.6, 16.3 |
| 2 | 1.9% | 1.0, 2.8 | 2.6% | 1.5, 3.7 | 0.1% | 0.0, 0.2 | 0.3% | 0.1, 0.4 | 0.0% | 0.0, 0.0 | 0.9% | 0.0, 1.8 | 2.3% | 1.0, 4.2 |
| 3 | 1.1% | 0.8, 1.5 | 1.4% | 0.8, 2.0 | 1.9% | 1.5, 2.4 | 2.2% | 1.8, 2.6 | 0.8% | 0.6, 1.0 | 0.4% | 0.2, 0.7 | 1.4% | 0.7, 2.5 |
| 4 | 2.3% | 1.7, 3.0 | 1.6% | 1.0, 2.1 | 3.2% | 2.6, 3.8 | 1.6% | 1.3, 1.9 | 5.7% | 4.7, 6.7 | 4.9% | 3.3, 6.6 | 1.8% | 1.0, 3.1 |
| 5 | 10.7% | 7.6, 13.8 | 6.7% | 4.5, 9.0 | 0.8% | 0.5, 1.1 | 8.5% | 7.2, 9.8 | 0.4% | 0.1, 0.7 | 2.8% | 1.5, 4.1 | 7.9% | 4.1, 13.5 |
| 6A | 9.4% | 7.2, 11.5 | 3.5% | 2.4, 4.6 | 4.4% | 3.8, 5.0 | 4.5% | 3.6, 5.4 | 3.6% | 2.9, 4.3 | 3.7% | 3.1, 4.3 | 5.3% | 3.0, 8.8 |
| 6B | 8.5% | 6.3, 10.7 | 11.5% | 9.0, 14.0 | 13.7% | 12.2, 15.3 | 9.4% | 8.4, 10.3 | 13.4% | 11.7, 15.1 | 12.0% | 9.3, 14.6 | 10.5% | 6.2, 16.8 |
| 7F | 0.8% | 0.4, 1.3 | 2.0% | 1.2, 2.8 | 3.2% | 2.4, 3.9 | 2.5% | 2.0, 3.1 | 1.0% | 0.7, 1.4 | 2.0% | 1.1, 2.8 | 1.7% | 0.8, 3.1 |
| 8 | 1.1% | 0.8, 1.5 | 0.6% | 0.3, 0.9 | 1.0% | 0.7, 1.3 | 0.8% | 0.4, 1.1 | 0.1% | 0.0, 0.2 | 0.9% | 0.4, 1.5 | 0.8% | 0.3, 1.5 |
| 9A | 0.4% | 0.2, 0.7 | 0.3% | 0.1, 0.5 | 0.1% | 0.1, 0.2 | 0.0% | 0.0, 0.1 | 0.4% | 0.2, 0.7 | 0.1% | 0.0, 0.2 | 0.3% | 0.1, 0.7 |
| 9V | 2.2% | 1.3, 3.1 | 3.1% | 2.2, 4.1 | 4.2% | 3.4, 5.1 | 2.7% | 2.3, 3.1 | 5.3% | 4.5, 6.0 | 3.9% | 3.1, 4.7 | 2.8% | 1.5, 4.9 |
| 12A | 0.1% | 0.0, 0.1 | 1.2% | 0.7, 1.8 | 0.0% | 0.0, 0.1 | 0.1% | 0.0, 0.1 | 0.0% | 0.0, 0.0 | 0.0% | 0.0, 0.0 | 0.8% | 0.3, 1.6 |
| 12F | 1.7% | 1.1, 2.3 | 1.6% | 0.8, 2.3 | 0.7% | 0.6, 0.9 | 0.6% | 0.3, 0.9 | 1.2% | 0.7, 1.7 | 2.2% | 0.9, 3.5 | 1.6% | 0.7, 2.9 |
| 14 | 13.0% | 10.0, 16.0 | 11.6% | 8.7, 14.5 | 23.9% | 21.0, 26.8 | 26.5% | 23.2, 29.7 | 29.2% | 26.4, 31.9 | 23.7% | 17.2, 30.1 | 12.9% | 7.6, 20.9 |
| 15B | 0.5% | 0.1, 0.9 | 0.8% | 0.4, 1.2 | 0.7% | 0.5, 0.8 | 0.7% | 0.4, 0.9 | 0.3% | 0.2, 0.4 | 0.2% | 0.0, 0.4 | 0.7% | 0.2, 1.4 |
| 18C | 1.4% | 0.9, 2.0 | 2.4% | 1.7, 3.2 | 6.9% | 5.9, 8.0 | 4.3% | 3.4, 5.2 | 8.0% | 6.9, 9.0 | 5.9% | 4.1, 7.7 | 2.3% | 1.2, 3.9 |
| 19A | 3.9% | 2.5, 5.3 | 2.6% | 1.7, 3.5 | 5.5% | 4.6, 6.4 | 2.9% | 2.3, 3.5 | 3.0% | 2.4, 3.7 | 3.9% | 2.9, 4.9 | 3.1% | 1.5, 5.4 |
| 19F | 5.4% | 3.6, 7.1 | 8.1% | 6.3, 9.8 | 8.2% | 7.1, 9.3 | 3.6% | 3.2, 4.1 | 10.3% | 9.3, 11.3 | 8.9% | 6.8, 11.0 | 7.1% | 4.1, 11.5 |
| 23F | 6.5% | 4.5, 8.5 | 9.7% | 7.6, 11.8 | 7.1% | 6.1, 8.2 | 5.3% | 4.4, 6.2 | 6.2% | 4.9, 7.5 | 5.2% | 3.7, 6.6 | 8.5% | 4.9, 13.7 |
| 45 | 0.5% | 0.0, 1.0 | 0.6% | 0.1, 1.0 | 0.0% | 0.0, 0.0 | 0.0% | 0.0, 0.0 | 0.0% | 0.0, 0.0 | 1.1% | 0.1, 2.1 | 0.5% | 0.1, 1.3 |
| 46 | 1.3% | 0.4, 2.1 | 0.5% | 0.1, 0.9 | 0.0% | 0.0, 0.0 | 0.0% | 0.0, 0.0 | 0.0% | 0.0, 0.0 | 1.0% | 0.0, 2.0 | 0.7% | 0.1, 1.6 |
| All Others | 15.7% | 12.7, 18.6 | 18.2% | 14.7, 21.6 | 9.2% | 7.9, 10.4 | 15.3% | 12.5, 18.1 | 10.2% | 7.0, 13.4 | 14.6% | 11.1, 18.1 | 17.1% | 10.5, 26.7 |
| TOTAL | 100.0% |  | 100.0% |  | 100.0% |  | 100.0% |  | 100.0% |  | 100.0% |  | 100.0% |  |
