# Supplementary material for: Systematic Evaluation of Serotypes Causing Invasive Pneumococcal Disease among Children Under Five: The Pneumococcal Global Serotype Project
Source: PLoS Med. 2010 Oct 5;7(10):e1000348. doi: 10.1371/journal.pmed.1000348 (PMC2950132; doi:10.1371/journal.pmed.1000348)
Supplement: Table S6 — Regional incidence of pneumococcal disease (per 100,000) in young children attributed to each serotype. Pneumococcal disease incidence was estimated by applying the proportion of pneumonia cases caused by SP derived from efficacy estimates from vaccine trials to WHO country-specific estimates of all-cause pneumonia cases then multiplied by country estimates of populations younger than 5 y of age (for more details on pneumococcal disease incidence estimates see [2]). LB, lower bound of uncertainty estimate; UB, upper bound of uncertainty estimate; SSI, serotype-specific incidence (per 100,000 children <5 y of age). (0.07 MB DOC) [file pmed.1000348.s014.doc]

**Table S6.**  Incidence of pneumococcal disease (per 100,000) in young children attributed to each serotype by region. LB = Lower bound of uncertainty estimate; UB = Upper bound of uncertainty estimate; SSI = Serotype-specific incidence (per 100,000 children <5 years of age). Pneumococcal disease incidence was estimated by applying the proportion of pneumonia cases caused by *S pneumoniae* derived from efficacy estimates from vaccine trials to WHO country-specific estimates of all-cause pneumonia cases then multiplied by country estimates of populations younger than 5 years of age (For more details on pneumococcal disease incidence estimates see: O'Brien KL, et al. (2009) Lancet 374: 893-902.)

| **Serotype** | **Africa** | | **Asia** | | **Europe** | | **Latin America and Caribbean** | | **North America** | | **Oceania** | | **Global** | |
| --- | --- | --- | --- | --- | --- | --- | --- | --- | --- | --- | --- | --- | --- | --- |
|  | SSI | LB, UB | SSI | LB, UB | SSI | LB, UB | SSI | LB, UB | SSI | LB, UB | SSI | LB, UB | SSI | LB, UB |
| 1 | 396 | 243, 582 | 232 | 126, 374 | 23 | 14, 35 | 98 | 65, 140 | 3 | 1, 5 | 21 | 9, 37 | 233 | 102, 451 |
| 2 | 65 | 25, 119 | 64 | 29, 113 | 0 | 0, 1 | 3 | 1, 6 | 0 | 0, 0 | 10 | 0, 26 | 52 | 17, 117 |
| 3 | 38 | 20, 61 | 35 | 16, 62 | 9 | 5, 13 | 25 | 16, 38 | 2 | 1, 3 | 5 | 1, 9 | 32 | 12, 68 |
| 4 | 79 | 43, 125 | 38 | 20, 63 | 15 | 9, 21 | 19 | 12, 28 | 16 | 10, 24 | 58 | 30, 94 | 43 | 18, 86 |
| 5 | 364 | 193, 584 | 164 | 84, 272 | 4 | 2, 6 | 100 | 65, 144 | 1 | 0, 2 | 32 | 14, 59 | 185 | 75, 374 |
| 6A | 317 | 182, 488 | 86 | 46, 139 | 20 | 13, 28 | 53 | 33, 79 | 10 | 6, 15 | 43 | 28, 62 | 125 | 54, 244 |
| 6B | 288 | 160, 451 | 280 | 169, 424 | 62 | 43, 87 | 109 | 76, 151 | 37 | 25, 53 | 140 | 85, 210 | 244 | 112, 462 |
| 7F | 28 | 10, 54 | 49 | 23, 86 | 14 | 9, 22 | 29 | 18, 45 | 3 | 1, 5 | 23 | 10, 41 | 39 | 14, 85 |
| 8 | 38 | 19, 63 | 15 | 6, 28 | 4 | 2, 7 | 9 | 4, 16 | 0 | 0, 1 | 11 | 3, 21 | 18 | 6, 40 |
| 9A | 15 | 6, 28 | 7 | 3, 14 | 1 | 0, 1 | 0 | 0, 1 | 1 | 0, 2 | 1 | 0, 3 | 8 | 2, 18 |
| 9V | 74 | 34, 129 | 76 | 41, 124 | 19 | 12, 29 | 31 | 20, 46 | 15 | 10, 21 | 46 | 29, 68 | 66 | 26, 135 |
| 12A | 2 | 0, 5 | 30 | 12, 55 | 0 | 0, 0 | 1 | 0, 2 | 0 | 0, 0 | 0 | 0, 0 | 19 | 6, 42 |
| 12F | 57 | 27, 99 | 38 | 16, 70 | 3 | 2, 5 | 7 | 3, 13 | 3 | 1, 6 | 26 | 8, 50 | 36 | 12, 80 |
| 14 | 441 | 254, 676 | 284 | 165, 440 | 108 | 74, 152 | 310 | 210, 436 | 81 | 57, 112 | 277 | 158, 435 | 302 | 137, 574 |
| 15B | 18 | 3, 39 | 19 | 7, 36 | 3 | 2, 5 | 8 | 3, 13 | 1 | 0, 1 | 3 | 0, 6 | 16 | 4, 39 |
| 18C | 48 | 22, 84 | 59 | 31, 96 | 31 | 21, 45 | 50 | 31, 76 | 22 | 15, 32 | 68 | 37, 110 | 53 | 21, 108 |
| 19A | 133 | 63, 226 | 64 | 33, 107 | 25 | 16, 36 | 34 | 21, 52 | 8 | 5, 13 | 45 | 27, 70 | 72 | 28, 149 |
| 19F | 182 | 92, 300 | 197 | 118, 299 | 37 | 25, 53 | 42 | 29, 59 | 29 | 20, 40 | 104 | 62, 159 | 164 | 73, 315 |
| 23F | 220 | 114, 359 | 237 | 143, 360 | 32 | 22, 46 | 62 | 39, 91 | 17 | 11, 26 | 60 | 34, 95 | 197 | 88, 378 |
| 45 | 17 | 0, 44 | 14 | 2, 31 | 0 | 0, 0 | 0 | 0, 0 | 0 | 0, 0 | 13 | 0, 31 | 12 | 1, 35 |
| 46 | 43 | 10, 90 | 12 | 2, 26 | 0 | 0, 0 | 0 | 0, 0 | 0 | 0, 0 | 12 | 0, 29 | 16 | 2, 44 |
| All Others | 532 | 322, 790 | 444 | 277, 658 | 41 | 28, 59 | 179 | 113, 266 | 28 | 15, 47 | 171 | 101, 262 | 399 | 189, 737 |
| TOTAL | 3,395 |  | 2,444 |  | 453 |  | 1,169 |  | 279 |  | 1,168 |  | 2,331 |  |
