# Supplementary material for: Systematic Evaluation of Serotypes Causing Invasive Pneumococcal Disease among Children Under Five: The Pneumococcal Global Serotype Project
Source: PLoS Med. 2010 Oct 5;7(10):e1000348. doi: 10.1371/journal.pmed.1000348 (PMC2950132; doi:10.1371/journal.pmed.1000348)
Supplement: Table S7 — Pneumococcal disease cases in young children attributed to each serotype by region. Pneumococcal disease cases were estimated by applying the proportion of pneumonia cases caused by SP derived from efficacy estimates from vaccine trials to WHO country-specific estimates of all-cause pneumonia cases among children younger than 5 y of age (for more details on pneumococcal disease cases estimates see [2]). LB, lower bound of uncertainty estimate; UB, upper bound of uncertainty estimate. (0.08 MB DOC) [file pmed.1000348.s015.doc]

**Table S7.**  Pneumococcal disease cases in young children attributed to each serotype by region. LB = Lower bound of uncertainty estimate; UB = Upper bound of uncertainty estimate. Pneumococcal disease cases were estimated by applying the proportion of pneumonia cases caused by *S pneumoniae* derived from efficacy estimates from vaccine trials to WHO country-specific estimates of all-cause pneumonia cases among children younger than 5 years of age (For more details on pneumococcal disease cases estimates see: O'Brien KL, et al. (2009) Lancet 374: 893-902.)

| **Serotype** | **Africa** | | **Asia** | | **Europe** | | **Latin America and Caribbean** | | **North America** | | **Oceania** | | **Global** | |
| --- | --- | --- | --- | --- | --- | --- | --- | --- | --- | --- | --- | --- | --- | --- |
|  | Cases | LB, UB | Cases | LB, UB | Cases | LB, UB | Cases | LB, UB | Cases | LB, UB | Cases | LB, UB | Cases | LB, UB |
| 1 | 519,819 | 318734, 764749 | 861,689 | 466853, 1392045 | 8,517 | 5167, 13017 | 54,600 | 36184, 78240 | 636 | 298, 1116 | 560 | 242, 1001 | 1,445,820 | 633578, 2800389 |
| 2 | 85,132 | 33287, 156700 | 238,303 | 106798, 420835 | 176 | 7, 430 | 1,758 | 521, 3567 | 0 | 0, 0 | 280 | 0, 705 | 325,651 | 107442, 725294 |
| 3 | 49,527 | 25745, 80551 | 130,554 | 59306, 229273 | 3,172 | 1887, 4909 | 14,138 | 8889, 21039 | 455 | 260, 723 | 126 | 38, 252 | 197,972 | 73105, 419338 |
| 4 | 103,514 | 56323, 164177 | 141,912 | 73079, 235377 | 5,362 | 3425, 7919 | 10,636 | 6697, 15810 | 3,437 | 2176, 5112 | 1,541 | 809, 2530 | 266,401 | 109658, 536321 |
| 5 | 477,627 | 253713, 767788 | 609,838 | 313013, 1013134 | 1,297 | 619, 2250 | 55,714 | 36432, 80633 | 229 | 25, 535 | 869 | 362, 1576 | 1,145,573 | 462999, 2321659 |
| 6A | 416,846 | 238735, 641296 | 318,286 | 170999, 516507 | 7,368 | 4958, 10480 | 29,437 | 18209, 44292 | 2,164 | 1358, 3237 | 1,156 | 757, 1662 | 775,256 | 333404, 1515378 |
| 6B | 378,010 | 210114, 592158 | 1,041,085 | 629597, 1576500 | 22,922 | 15858, 31909 | 61,159 | 42569, 84330 | 8,109 | 5451, 11546 | 3,740 | 2288, 5633 | 1,515,025 | 693392, 2865179 |
| 7F | 36,763 | 12682, 70483 | 182,554 | 84391, 318242 | 5,259 | 3138, 8122 | 16,447 | 9895, 25199 | 624 | 318, 1053 | 618 | 280, 1085 | 242,265 | 85289, 527323 |
| 8 | 50,211 | 25330, 82947 | 56,099 | 22194, 103807 | 1,644 | 867, 2722 | 4,932 | 2004, 9120 | 53 | 0, 145 | 289 | 93, 566 | 113,228 | 38768, 248364 |
| 9A | 19,409 | 7263, 36266 | 27,658 | 9383, 53685 | 208 | 75, 399 | 272 | 64, 579 | 262 | 84, 522 | 36 | 1, 87 | 47,845 | 13292, 113383 |
| 9V | 97,799 | 44622, 169401 | 282,873 | 150829, 460878 | 7,028 | 4386, 10546 | 17,580 | 11453, 25514 | 3,175 | 2099, 4578 | 1,228 | 771, 1819 | 409,683 | 163933, 836877 |
| 12A | 2,855 | 532, 6228 | 111,640 | 45554, 204351 | 49 | 0, 135 | 330 | 0, 847 | 0 | 0, 0 | 0 | 0, 0 | 114,875 | 35445, 262761 |
| 12F | 75,509 | 34950, 129962 | 142,400 | 58660, 259765 | 1,223 | 728, 1892 | 3,738 | 1474, 6986 | 721 | 322, 1292 | 691 | 225, 1353 | 224,283 | 74213, 498527 |
| 14 | 579,101 | 333601, 887692 | 1,055,778 | 613895, 1638266 | 39,900 | 27338, 55971 | 172,913 | 117125, 243668 | 17,646 | 12362, 24310 | 7,409 | 4225, 11643 | 1,872,746 | 849571, 3561677 |
| 15B | 23,439 | 4141, 51498 | 70,276 | 24409, 135494 | 1,088 | 626, 1716 | 4,216 | 1955, 7404 | 161 | 70, 292 | 73 | 7, 171 | 99,253 | 24368, 244764 |
| 18C | 63,515 | 28566, 110703 | 218,637 | 116053, 357064 | 11,523 | 7613, 16616 | 27,903 | 17068, 42296 | 4,810 | 3242, 6833 | 1,832 | 996, 2956 | 328,220 | 132919, 667702 |
| 19A | 174,157 | 82552, 296523 | 239,569 | 122187, 399248 | 9,179 | 5966, 13394 | 19,091 | 11727, 28860 | 1,828 | 1115, 2787 | 1,217 | 711, 1886 | 445,041 | 171686, 925064 |
| 19F | 239,332 | 121304, 394420 | 731,840 | 439930, 1112475 | 13,697 | 9270, 19398 | 23,545 | 15948, 33182 | 6,237 | 4365, 8598 | 2,787 | 1660, 4268 | 1,017,438 | 454138, 1956313 |
| 23F | 289,472 | 149605, 472251 | 882,081 | 532380, 1337424 | 11,897 | 7934, 17038 | 34,571 | 22038, 50957 | 3,737 | 2284, 5693 | 1,615 | 910, 2556 | 1,223,374 | 548289, 2346855 |
| 45 | 22,897 | 0, 58040 | 52,058 | 7700, 117055 | 0 | 0, 0 | 0 | 0, 0 | 0 | 0, 0 | 341 | 11, 823 | 75,296 | 5538, 219568 |
| 46 | 56,173 | 12806, 118627 | 43,722 | 6383, 98447 | 0 | 0, 0 | 0 | 0, 0 | 0 | 0, 0 | 315 | 2, 774 | 100,211 | 14400, 271760 |
| All Others | 699,056 | 422910, 1037960 | 1,651,039 | 1031635, 2446835 | 15,272 | 10247, 21770 | 99,880 | 62894, 148482 | 6,178 | 3282, 10214 | 4,574 | 2716, 7019 | 2,476,000 | 1175221, 4569529 |
| TOTAL | 4,460,164 |  | 9,089,893 |  | 166,781 |  | 652,861 |  | 60,461 |  | 31,297 |  | 14,461,457 |  |
