# Supplementary material for: Systematic Evaluation of Serotypes Causing Invasive Pneumococcal Disease among Children Under Five: The Pneumococcal Global Serotype Project
Source: PLoS Med. 2010 Oct 5;7(10):e1000348. doi: 10.1371/journal.pmed.1000348 (PMC2950132; doi:10.1371/journal.pmed.1000348)
Supplement: Table S10 — The number of studies in our analysis with serotype data from children age ≥60 mo, by region. (0.03 MB DOC) [file pmed.1000348.s018.doc]

**Table S10.** The number of studies in our analysis with serotype data from children age 60+months, by region.

| Region | No. studies including isolates from children age ≥ 60 months | No. studies including isolates from children age ≥72 months |
| --- | --- | --- |
| Africa | 9/22 | 9/22 |
| Asia | 16/33 | 14/33 |
| Europe | 8/39 | 2/39 |
| Latin America and Caribbean | 24/42 | 4/42 |
| North America | 3 /17 | 0/17 |
| Oceania | 3/16 | 2/16 |
